# Hot spot prediction in protein-protein interactions by an ensemble system

**DOI:** 10.1186/s12918-018-0665-8

**Published:** 2018-12-31

**Authors:** Quanya Liu, Peng Chen, Bing Wang, Jun Zhang, Jinyan Li

**Affiliations:** 1 Institute of Physical Science and Information Technology, Anhui University, Hefei, Anhui, 230601 China; 2 School of Electrical and Information Engineering, Anhui University of Technology, Ma’anshan, Anhui, 243032 China; 3 School of Electrical and Information Engineering, Anhui University of Technology, Ma’anshan, Anhui, 243032 China; 4 School of Electrical Engineering and Automation, Anhui University, Hefei, Anhui, 230601 China; 5 Advanced Analytics Institute and Centre for Health Technologies, University of Technology, Sydney, Sydney, Broadway, NSW, 2007 Australia

**Keywords:** Hot spot residues, Protein-protein interaction, Ensemble learning

## Abstract

**Background:**

Hot spot residues are functional sites in protein interaction interfaces. The identification of hot spot residues is time-consuming and laborious using experimental methods. In order to address the issue, many computational methods have been developed to predict hot spot residues. Moreover, most prediction methods are based on structural features, sequence characteristics, and/or other protein features.

**Results:**

This paper proposed an ensemble learning method to predict hot spot residues that only uses sequence features and the relative accessible surface area of amino acid sequences. In this work, a novel feature selection technique was developed, an auto-correlation function combined with a sliding window technique was applied to obtain the characteristics of amino acid residues in protein sequence, and an ensemble classifier with SVM and KNN base classifiers was built to achieve the best classification performance.

**Conclusion:**

The experimental results showed that our model yields the highest F1 score of 0.92 and an MCC value of 0.87 on ASEdb dataset. Compared with other machine learning methods, our model achieves a big improvement in hot spot prediction.

**Availability:**

http://deeplearner.ahu.edu.cn/web/HotspotEL.htm.

**Electronic supplementary material:**

The online version of this article (10.1186/s12918-018-0665-8) contains supplementary material, which is available to authorized users.

## Background

Protein is one of important biological macro-molecules in organisms. Protein-protein interactions play a mediating role in protein function biologically [1]. In order to better understand the mechanism of protein-protein interactions, hot spot residues have to be studied. By studying hot spot residues, small molecules that bind to hot spot residues can be designed to prevent erroneous protein-protein interactions [2]. On the other hand, the study of hot spot residues can also be used to predict the secondary structure of proteins. Saraswathi et al. found that different amino acid distributions play a crucial role in determining secondary structures [3]. In previous studies, hot spot residues were identified by experimental methods, such as alanine mutagenesis scanning [4]. Based on the large number of mutations created by experimental methods, relevant researchers can extract a large number of accurate hot spot residues and apply them to investigate functional sites of protein-protein interactions [5]. With the increase of mutation data, researchers established many standard databases focused on hot spot residues, such as binding interface database (BID) [6] and Alanine Scanning Energetics database (ASEdb) [7]. However, experimental methods are time-consuming and laborious to keep up with the speed of increasing demand for research data. Machine learning methods can be used to alleviate the disadvantages of experimental methods and identify hot spot residues.

Feature selection is an important part of developing prediction method. With the popularity of big data, researchers have developed multiple websites for feature extraction and selection. Our previous work proposed a new sequence-based model that combines physicochemical features with the relative accessible surface area of amino acid sequences for hot spot prediction [8]. Bin Liu et al. developed a python package that can extract features and implement model training [9], which can be used to identify post-translational modification sites and proire-protein binding sites. In addition, they also proposed a server that can generat pseudo components of biological samples, such as protein and DNA [10], which yields different outputs for different modes, including sequence types, heat vectors between feature vectors and feature vectors. Furthermore, some researchers have suggested that websites dedicated to feature selection can be used for different models. Chen et al. proposed a Python package for feature extraction and selection [11], which properly processes the sequence and structural characteristics of proteins and peptides, making these features more suitable for training model.

Many machine learning methods have been developed to identify hot spot residues. Some of them determined hot spot residues by calculating the energy contribution of each interfacial residue during protein-protein interactions such as Robetta server [12]. It is worth noting that most of the machine learning methods tried to train data with extracting relevant features from the sequence or structure information of proteins, and then test on unknown hot spot data. For example, *β* ACV _*ASA*_ integrated water exclusion theory into *β* contacts to predict hot spots [13]. Other methods used structure-based calculations to predict hot spot residues. Wang et al. proposed a novel structure-based computational approach to identify hot spot residues by docking protein homologs [14]. Furthermore, Xia et al. proposed APIS model based on structural features and amino acid physicochemical characteristics, and used SVM to train the model [15]. The classification model worked well and yielded an F1 score of 0.64. In addition, some researchers developed network methods to predict hot spots. Ye et al. used residue-residue network features and micro-environment feature in combination with support vector machines to predict hot spots, which yielded an F1 score value of 0.79 [16]. Although many methods have been developed to predict hot spots, the prediction performance is still low and the used structural features is difficult to obtain. Therefore, it is important for us to improve hot spot prediction and find more effective features.

Ensemble learning methods have been applied in various research fields. It is divided into feature fusion and decision fusion, which can combine the advantages and avoid the disadvantages of different classifiers, thus optimize model and improve classification accuracy. For example, He et al. developed an ensemble learning for face recognition, which used KNN and SVM training features with weighted summation decision matrices to obtain the optimal ensemble classifier. In general, combining multi-classifiers performs better than single classifier [17]. For example, Pan et al. used integrated GTB(Gradient Tree Boosting), SVM and ERT(Extremely Randomized Trees) to predict hot-spot residues between proteins and RNA, which yielded an ACC of 0.86 [18].

In order to address the above issues in hot spot predictions, this paper proposed a novel ensemble machine learning system with feature extraction to identify hot spot residues. The method is based on protein sequence information alone. First, our method obtained 46 independent amino acid sequence properties from AAindex1 [19] and relative accessible surface area (relASA) [20] from NetSurfP website to encode protein sequence. Then, the method combined an auto-correlation function with sliding window to encode these properties into amino acid features. Last, a new ensemble classifier, which combined the k-Nearest-Neighbours (KNN) [21] and SVM with radial basis Gaussian function [22], was built to train and test the curated data sets. Here, the publicly available LIBSVM software [23] was used to predict hot spot residues. As a result, our model achieved good prediction performance on different data sets. On the **ASEdb training set**, our method achieved the highest F1 value of 0.92 and an MCC value of 0.87 than state-of-the-art methods.

## Methods

### Data sets

There are many definitions of hot spot residues in previous studies. In alanine mutant scanning experiments, hot spot is defined as the residue whose change value of binding free energy is greater than 2 Kcal/mol, and non-hot spot residue with less than 0.4 Kcal/mol, while the rest ones are unnecessary, when the interface residues on PPIs are mutated to alanine [24]. It has been confirmed that most of the previous researchers used the criterion [25]. The ratio of positive instances to negative ones under this definition is basically close to 1, which is more credible when using the criterion for training one model [25]. According to this definition, two data sets were used in this work, the train set from Alanine Scanning Energetics Database (ASEdb) and the test set from binding interface database (BID). The data in the two databases are all verified by alanine mutation scan experiments. The BID data set is divided into four sub-groups: ’strong’, ’intermediate’, ’weak’ and ’insignificant’ interactions. Here, those residues labeled with ’strong’ are considered as hot spots and the rest residues are non-hot spots for our model.

In this study, ten-fold cross validation method was adopted to train our model and test on BID data. In order to verify the effectiveness of the model, three independent test sets were applied. The first one was SKEMPI (Structural Kinetic and Energetic database of Mutant Protein Interactions), which contains a lot of mutant data from scientific literature. Actually, a small amount of alanine mutation data was used in this database [26]. The second one was dbMPIKT (the kinetic and thermodynamic database of mutant protein interactions), which is a database of mutated proteins that we have collected from scientific literature in recent years [27]. The last one is a mixed set of the former datasets, where the same items in the two independent test sets were removed. In addition, protein sequences in each database must have a sequence identity less than 35% after the removal of redundancy and homology bias. Detailed description of the data sets is shown in Table 1.

**Table 1:** Databases for hot spots prediction

Data sets	Positive sample(HS)	Negative sample(NHS)	Total
Train set(ASEdb)	58	91	149
Test set(BID)	70	115	185
Independent test(SKEMPI)	120	234	354
Independent test(dbMPIKT)	106	384	490
Independent test(Mix set)	292	697	989

### Ensemble learning method

#### Feature selection

To identify whether residues are hot spots, protein sequences have to be encoded into numerical sequences. To better characterize protein sequences, AAindex1 database was used, which contains 544 physicochemical and biochemical properties for 20 types of amino acids. Since highly related properties may make the predictions bias, relevant ones with a correlation coefficient more than 0.5 were removed in this work [28]. First, the correlation coefficients, *CCp*_*i*_, between a property, *p*_*i*_, *i*=1-544, and the other ones are calculated. Then the number of relevant properties, *Np*_*i*_, is counted for the property *p*_*i*_. The calculation is repeated for all of the 544 properties. After the process, 46 properties were obtained and used to characterize protein sequences. The details of the properties are listed in Additional file 1.

In order to reflect the importance of the order of residues in protein sequence, auto-correlation function was used to calculate the attribute correlation coefficient of one residue and its neighbor residues in protein sequence as a one-dimensional feature [29].

The auto-correlation function *r*_*j*_ is defined as:

1 $$\begin{array}{@{}rcl@{}} r_{j} = \frac{1}{L-1}\sum\limits_{l=1}^{L-j} h_{l}*h_{l+j}, j= 1,2,3,.....M, \end{array} $$

where *h*_*l*_ is one amino acid property for the *l*-th residue, *L* is the length of protein sequence and the *M* value is the number of neighbors that needs to be adjusted.

**Fig. 1:**
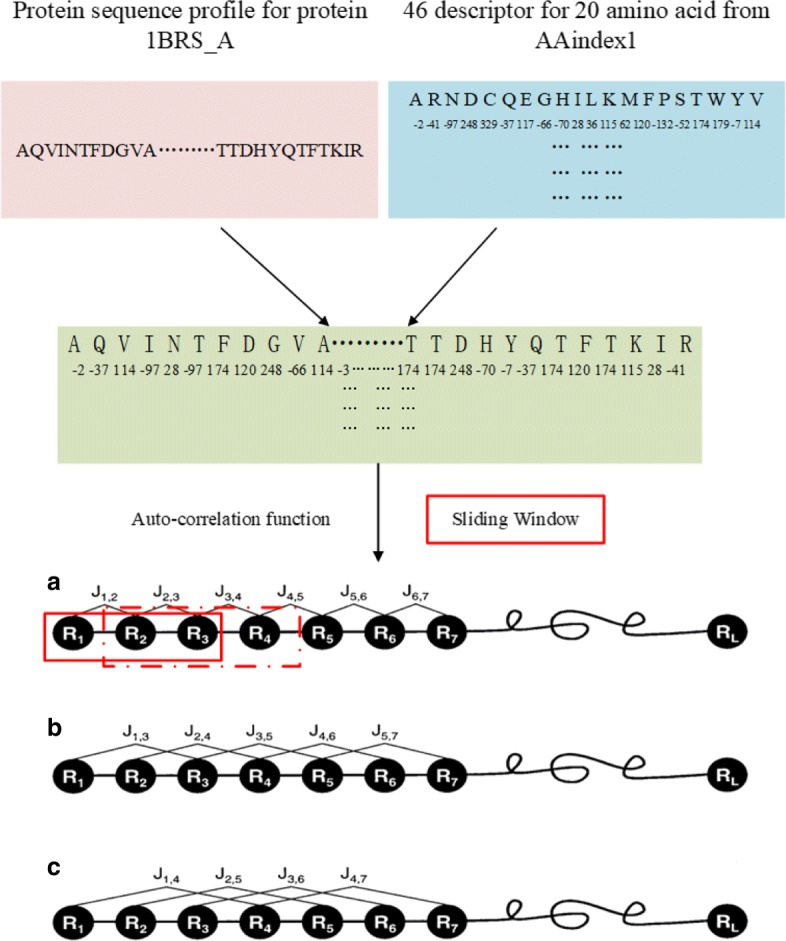
Encoding schema for protein residues. The protein sequence was first converted to a numerical sequence using the 46 attributes of AAi2dex1. Then, each residue is encoded using the autocorrelation function combined with the sliding window. Here, *R*_*1*_ represents the 1st residue in the protein sequence, *R*_*2*_ represents the 2nd residue..., and *R*_*L*_ represents the *L*-th residue, each of them belongs to the 20 common types of amino acids

To investigate the detailed role of amino acid in the entire protein sequence, the auto-correlation function and the property of each amino acid were integrated into the encoding schema. Moreover, the sliding window was used to calculate the auto-correlation coefficient of the protein sequence in segments, and the auto-correlation coefficient of each amino acid was obtained for each property. Every residue in a protein sequence was encoded by a set of sequential auto-correlation coefficients derived from its neighbor residues. Let’s set *L* be the length of the sliding window, select a residue as the center residue and calculate the correlation coefficient of the center residue using the correlation coefficient between residues around the central one in the window. Especially, it is worth noting that the value of the void place is set to one when the distance of the central residue and the end of the sequence is less than *L/2*. As a result,*(L-1)/2* features can be obtained to represent each central residue. The details of the encoding schema can be seen in Fig. 1.

In addition, the ASA value of each residue can be calculated by web server NetsurfP (http://www.cbs.dtu.dk/services/NetSurfP/) and then used as a feature in this work [30]. In total, every residue is represented by an input vector with 46*L features.

#### Classifier construction

**Fig. 2:**
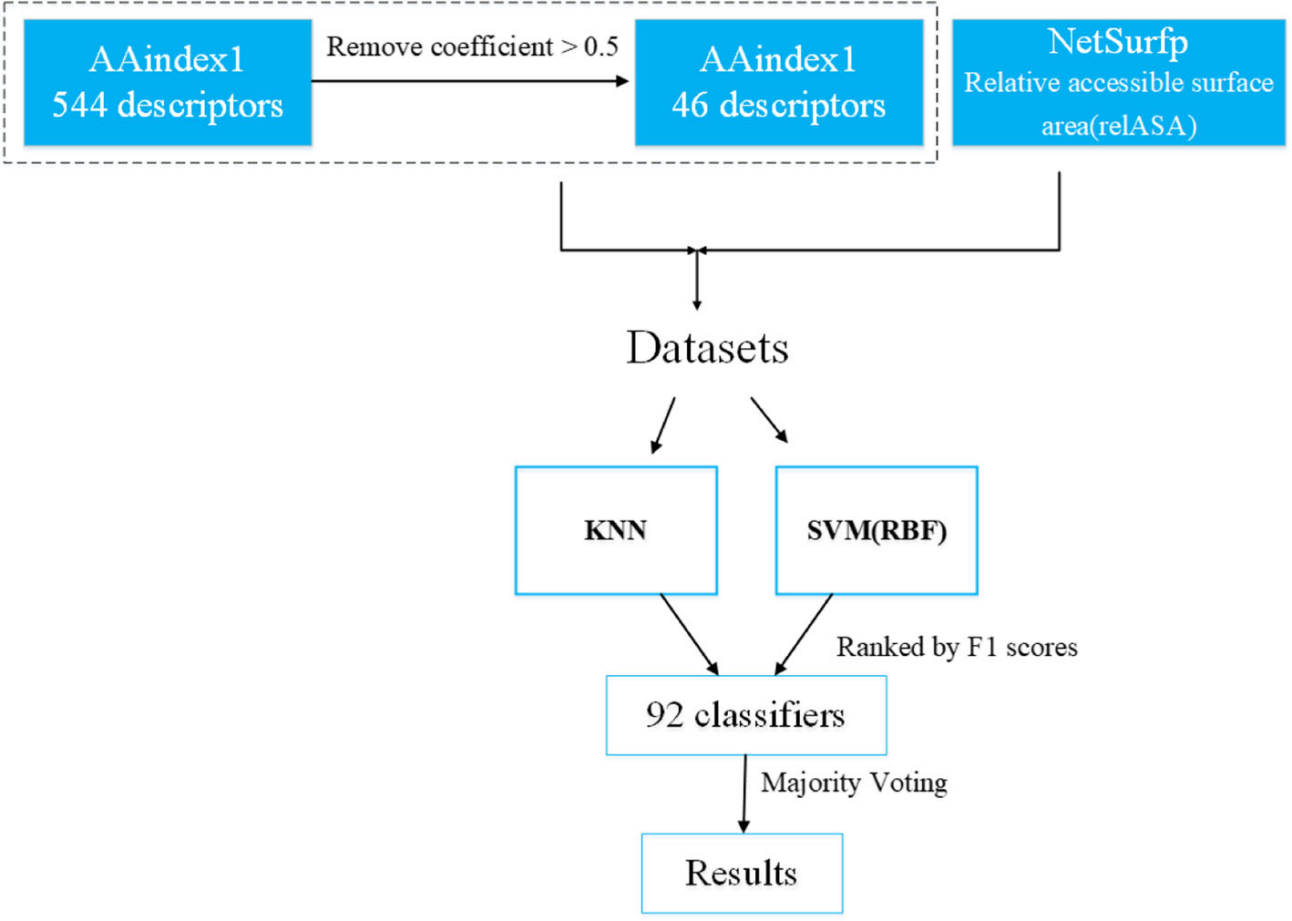
The flowchart of our model

Since the datasets used in this work are much small, ensemble machine learning method was proposed. Ensemble learning is more popular in the current machine learning field, it can integrate the advantages of different classifiers and create models with good classification performance [31]. KNN classifier and SVM classifier are chose as the base classifiers of the ensemble learning method. In the field of machine learning, support vector machines have great advantages and good generalization ability in problems with small sample datasets, and KNN is based on statistically established classifier algorithm [25]. Therefore, the two types of KNN and SVM have chosen. The KNN-SVM joint classifier can make up for the shortcomings between the two classifiers and thus improve the classification accuracy [32]. Based on the 46 descriptors from AAindex1, residue encoding vector with each descriptor is regarded as an input into KNN and SVM training models. Then, the outputs of all classifiers are sorted in terms of F1 scores. Moreover, majority voting is applied to integrate the classifiers and the combination of the top *n* classifiers is explored. Here, top *n* classifiers are chosen in that the classification performance of the ensemble learner is the best. In addition, the flowchart of our model is shown in Fig. 2 and the implementation of the mothed in MATLAB can be referred to Additional file 2.

### Evaluation criteria

There are many metrics to evaluate the quality of machine learning model. Some of the most commonly used ones include accuracy (ACC), specificity (SPE), recall, F1 score (F1) and Matthews correlation coefficient (MCC). Furthermore, the Receiver Operating Characteristic (ROC) curves and area under ROC curve (AUC) values can be also used as evaluation criteria. Among them, F1, MCC and AUC are the important metrics to comprehensively evaluate models [33, 34].

In this study, confusion matrix was adopted to calculate evaluation index [35]. Especially, four values in the confusion matrix, TP, FP, TN and FN, respectively, represent the number of true positives (correctly predicted hot spots), the number of false positives (incorrectly predicted hot spots), the number of true negatives (correctly predicted non-hot spots) and the number of false negatives (incorrectly predicted non-hot spots). Specific calculation formula are shown in Eq. (2).

2 $$ {\begin{aligned} ACC &=\frac{TP+TN}{TP+FP+TN+FN} \\ PRE &= \frac{TP}{TN+FP} \\ SEN &= \frac{TP}{TP+FN} \\ F1 &= \frac{2*SEN*PRE}{SEN+PRE} \\ MCC&=\frac{TP*TN-FP*FN}{\sqrt{(TP\,+\,FP)(TP\,+\,FN)(TN\,+\,FP)(TN\,+\,FN)}} \end{aligned}}  $$

## Results

### Performance of ensemble classifiers on different *M* for auto-correlation function

**Fig. 3:**
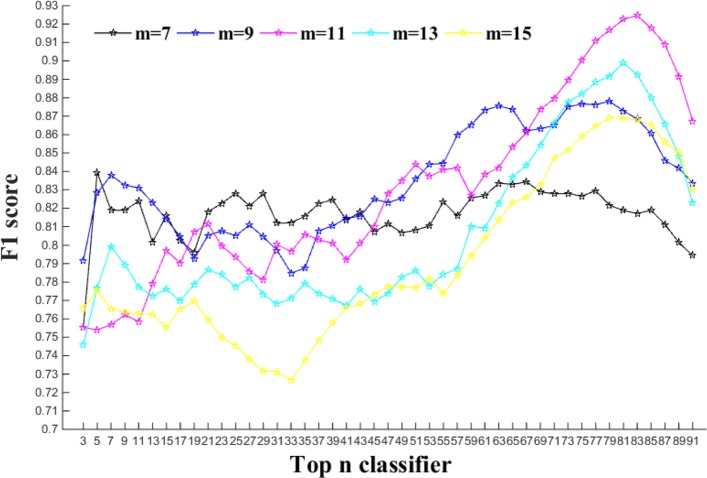
Performance comparison of the model with different m values

The experiments of our model are trained by ten-fold cross validation on the ASEdb train set. That is to say, during the training process, the dataset is randomly divided into ten subsets with roughly the same number of samples, nine of them are taken as training data and the other one is used as test data. The concatenation of the ten outputs of experiments yields the whole training outputs. In the training process, when we use autocorrelation function, the value of *M* needs to be adjusted that directly determines the dimension of encoding feature vectors and also affects the classification performance. Considering the problem with too high feature dimension, a smaller range of *M* values has to be chosen. The classification effect is normally distributed by the selection of different *M* values, which has to be chosen to make the model yielding good prediction performance. In this study, the model with five *M* values was investigated. The performance of the model with different m values are shown in Fig. 3. It can be seen from the Fig. 3 that the model achieves the best F1 score on ASEdb when the *M* value is 11. Therefore, the dimension of encoding vectors is set as 46*11.

### Performance of different classifiers on ASEdb

**Fig. 4:**
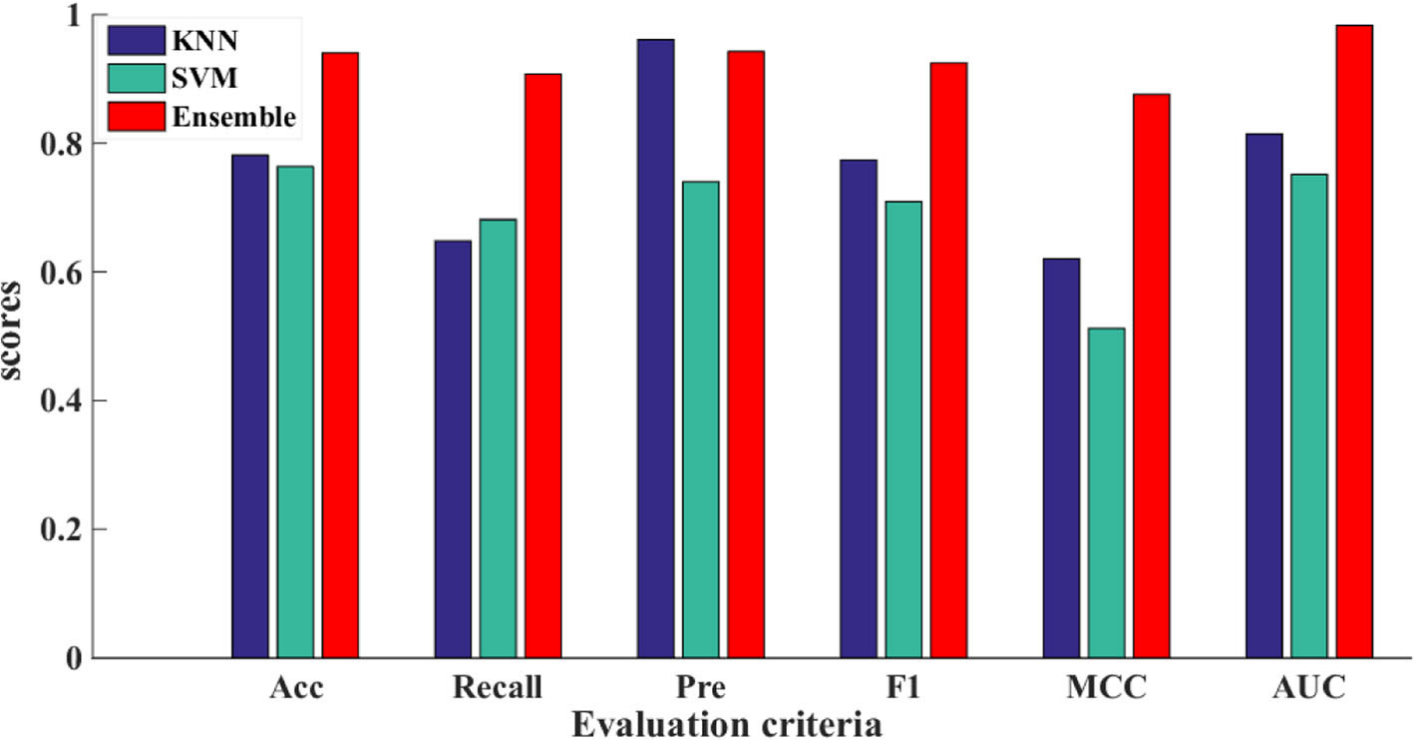
Performance comparison of three classifiers on ASEdb

Our proposed method is an ensemble learner, whose base classifiers are KNN and SVM. In order to highlight the advantages of the ensemble classifier, the performance comparison with classifiers of KNN and SVM was also investigated. The latter ones were implemented by default parameters. Figure 4 shows the performance comparison of the three classifiers. From the Fig. 4, it can be seen that our ensemble classifier outperforms individual KNN classifier and individual SVM classifier, while KNN outperforms SVM in metrics of Pre, F1, AUC and ACC. Our method achieves an ACC value of **0.94**, an AUC value of **0.98** and an F1 scores of **0.92**. In summary, our ensemble classifiers model works well for predicting hot spot residues.

### Performance of our model on train and test sets

**Fig. 5:**
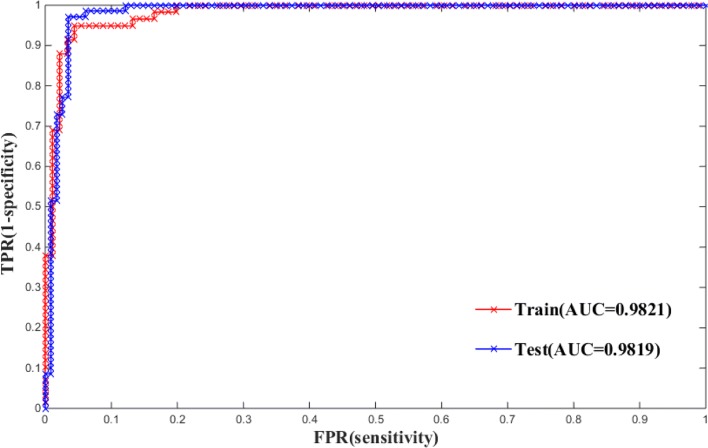
The ROC curves of the ensemble model with the top 83 classifiers on training and test sets

After determining required parameters, the ensemble system was trained on ASEdb by ten-fold cross-validation, then tested on BID to obtain the prediction performance of the model. To obtain good performance, ensemble model with different numbers of base classifiers from 3 to 91 was investigated. The aim is to find out the best number of base classifiers for the ensemble model. Figure 3 demonstrates F1 scores of ensemble model with different combinations of the top *n* classifiers, where *n* is in the range of 3-91 in this study. As a result, the model with top 83 base classifiers yields the highest F1 score, whose prediction performance on training and test sets are shown in Table 2. From Table 2, it can be seen that the model yields a good classification performance on the training set and test set. In order to comprehensively evaluate the classification performance of the model, ROC curves of the model are illustrated in Fig. 5 and the corresponding AUC values are calculated on the training and test set.

**Table 2:** Prediction performance of top 83 classifier on training and test sets

Data sets	ACC	SPE	RECALL	PRE	F1	MCC
Train(ASEdb)	0.9402	0.9627	0.9078	0.9426	0.9247	0.8759
Test(BID)	0.9150	0.9595	0.8471	0.9476	0.8941	0.8278

**Fig. 6:**
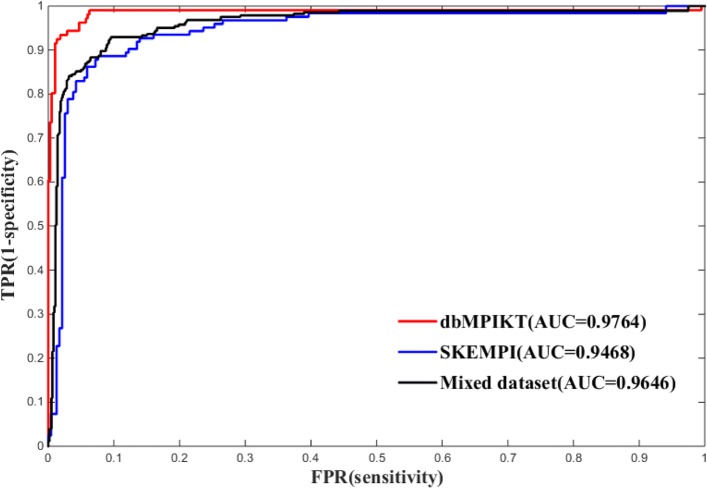
The ROC curves of the ensembles of the top 83 classifiers for SKEMPI, dbMPIKT and Mix sets

In order to verify the model’s generalization capability, three independent sets were applied to test the model. Table 3 lists the performance comparison of the three test datasets. Obviously, the ensemble model on mixed test sets yields an slightly higher F1 score of 0.8657 than that on the other two test sets. There may be two reasons for the slight difference. One is that the numbers of data sets are different, and the other is the different proportions of the positive and negative samples for the three test datasets. To sum up, our model has good performance on different data sets and is applicable to other data sets. Moreover, ROC curves and AUC values of the model for different test sets were also investigated. Figure 6 shows the ROC curves of the ensemble model with top 83 base classifiers. The AUCs (area under ROC curve) are 0.9468, 0.9764 and 0.9646 for dbMPIKT, SKEMPI and Mixed dataset, respectively. It can be concluded that our ensemble model yielded good performance for different test datasets.

**Table 3:** Prediction performance of model with top 83 classifiers on different test sets

Data sets	ACC	SPE	RECALL	PRE	F1	MCC
Test(SKEMPI)	0.9028	0.9268	0.8573	0.8590	0.8579	0.7843
Test(dbMPIKT)	0.9322	0.9616	0.8364	0.8618	0.8472	0.8052
Test(Mix set)	0.9183	0.9491	0.8503	0.8802	0.8644	0.8069

### Comparison with other methods

Several machine learning methods have developed to predict hot spots. Based on BID, as independent test dataset, our model was compared with five methods, Hot Point [36], PPRF [37], HEP [38], PredHS [39] and Hu’method [40]. The prediction comparison of these methods is shown in Table 4. Among the six methods, our model achieves the highest F1 score of 0.89 and an highest MCC of **0.83**, while the other three methods achieves an F1 value of 0.80 and an MCC of 0.65. All in all, our model performs better than other previous methods in hot spot prediction.

**Table 4:** Prediction comparison of different methods on BID test sets

Method	Features	ACC	F1	PRE
Hot point	Structural features	0.72	0.49	0.55
ppRF	B-factor, individual atomic contacts and the co-occurring contacts	0.78	0.58	0.69
HEP	Physicochemical, structural neighborhood features	0.79	0.70	0.60
PredHS	Structural neighborhood features	0.88	0.76	0.79
Hu method	Sequence features	0.76	0.80	1.0
Our method	Sequence features	0.92	0.89	0.95

## Discussion

### Feature selection algorithm

In this work, the auto-correlation function was chosen as the feature selection algorithm. The auto-correlation function takes into account not only the characteristic properties of amino acids but also the position information of amino acids in protein sequence and the influence between adjacent amino acids [41]. In order to verify the advantages of choosing the algorithm, the adopted feature selection algorithm was compared with other algorithms, where Hu’s feature selection algorithm selected pseudo-amino acid composition. The two algorithms were respectively applied as feature selection and then ran the ensemble learning model. The performance comparison is shown in Table 5. As shown in the Fig. 5, our method performs better than hu’s feature selection method with an improvement of 0.029 in F1 measure.

**Table 5:** Comparison of performance under different feature selection on training set

Data sets	ACC	SPE	RECALL	PRE	F1	MCC
Our method	0.9402	0.9627	0.9078	0.9426	0.9247	0.8759
Hu’s feature selection	0.9262	0.8959	0.9918	0.8174	0.8956	0.8494

### Feature correlation analysis

**Fig. 7:**
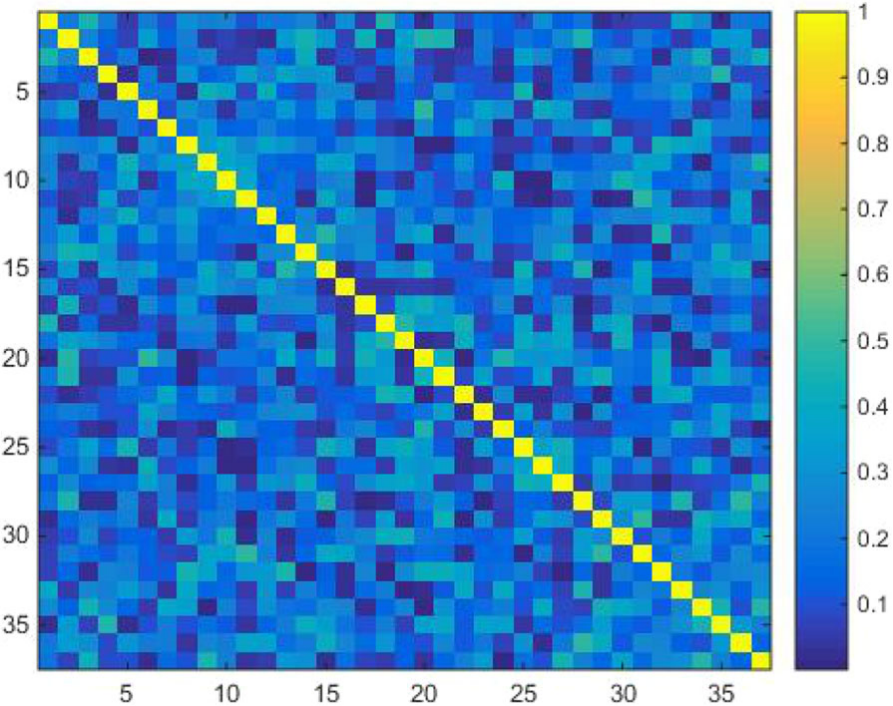
Correlation coefficient heat map of 37 features

In order to further study our model, we counted the number of base classifiers used in the model and the number of features. The statistical results are shown in Table 6. For KNN, all features are selected in our model, and only 37 features are selected for SVM. Next, we conducted a correlation analysis of the shared features and the heat map is shown in Fig. 7. From Fig. 7, it is obvious that the correlation of all features is basically less than 0.4, and some features are negatively correlated. This indirectly shows that the features selected in our model have a certain classification effect, and there is no redundancy between features.

**Table 6:** The classification and quantity statistics of base classifiers

Classifier	Number	Features
KNN	46	1-46
SVM(RBF)	37	1-3, 5-14, 18-31, 33, 34, 36, 37, 39, 40, 43-46

^*^The feature corresponds to the feature number in Additional file 1

### Descriptor cluster analysis

**Fig. 8:**
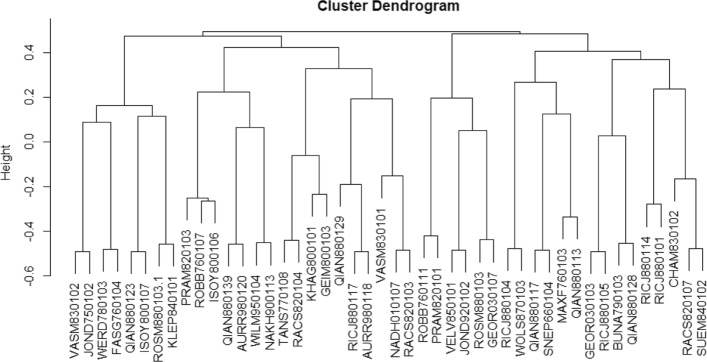
The cluster dendrogram of the 46 descriptors

As we all known, our features were created from AAindex1, which are all the characteristics of protein sequence. According to the original classification of AAindex1, the characteristics of our selected descriptors are divided into six groups. Classification results for AAindex1 properties are shown in Table 7. Especially, most of these descriptors are Alpha and Turn propensities, which is a conformational index of amino acids. The amino acid conformational bias can affect the secondary structures of protein interaction interface, and the frequency of occurrence of amino acids in different secondary structures is also different [42]. A few descriptors are physcioemcial properties, such as pH. In addition, all of the 46 descriptors have been completely clustered in the hierarchy [43]. The cluster dendrogram is shown in Fig. 8, where the abscissa represents descriptor and the ordinate represents the distance between two descriptors at custering. The distance is the correlation coefficient between descriptors. When one ordinate value is negative, it indicates that the two descriptors are negatively correlated. It can be seen from the tree diagram that the distances between attributes of different categories reflect the non-redundancy of the selected attributes in this work.

**Table 7:** The classification and quantity statistics of AAindex1 properties

Alpha and Turn propensities	GEIM800103, CHAM83102, QIAN880129, ROBB760111, RICJ880114
	RACS820104, QIAN880117, WOLS870103, FASG760104, ISOY800106
	ROBB760107, QIAN880139, QIAN880113, RICJ880117, SNEP660104
	VASM830101, BUAN790103
Hydrophobicity	NAKH900113, QIAN880128, PRAM820101, KHAG800101, SUEM840102
	WERD780103, RICJ880104, VASM830102, ROSM880103, RICJ880105
	ISOY800107, RACS820103, JOND750102, TANS770108, KLEP840101, VELV850101
Physcioemcial properties	JOND920102, QIAN880113
Add properties	GERO01103, NADH010107, AURR980118, AURR980120, WILM950104
	GEOR030107, GERO01103

^*^The second column represents the number of each attribute in AAindex1

### Case study

**Fig. 9:**
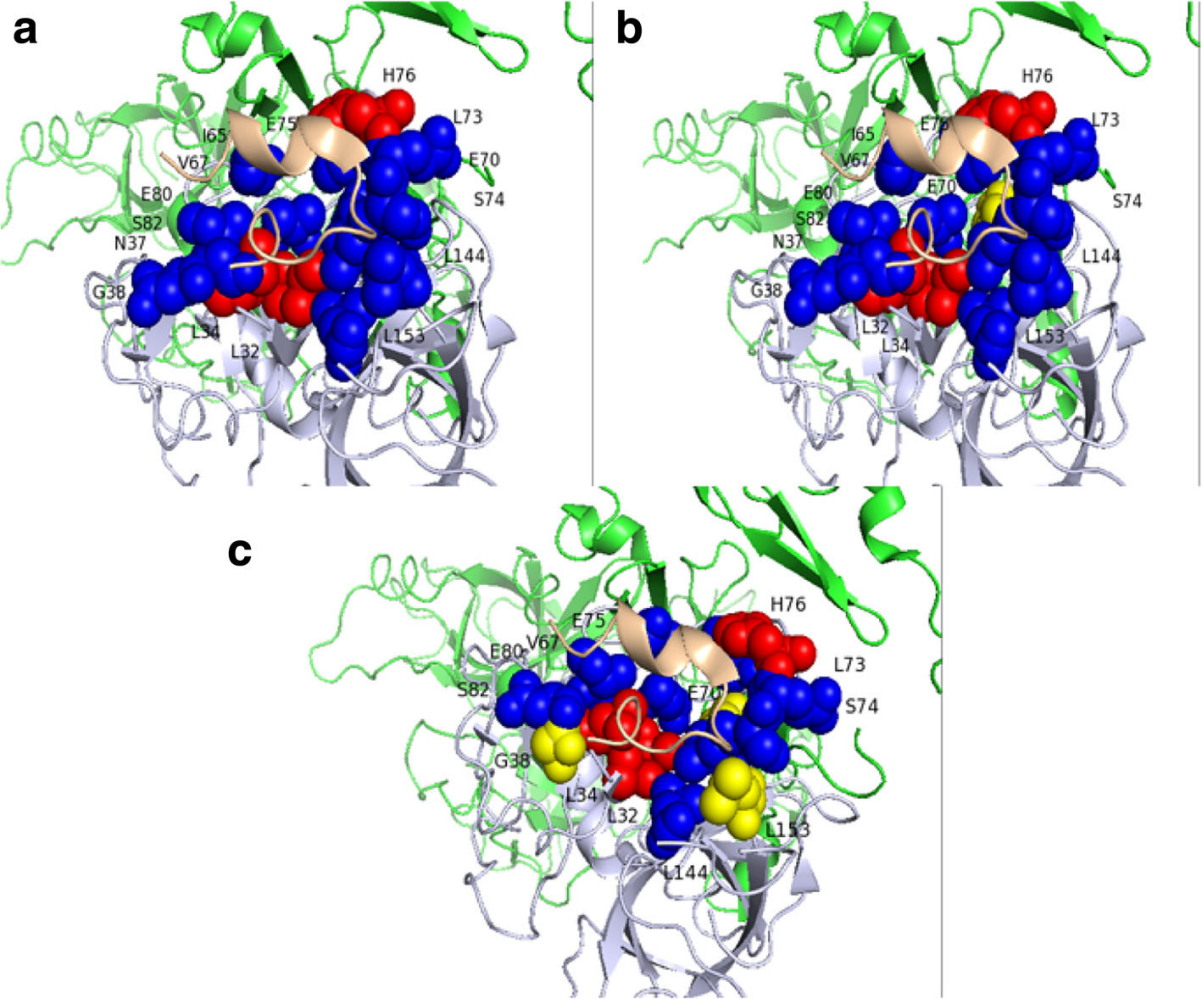
The visualization of prediction performance for PDB ID: 1DVA(chain H and chain X). Hot spots are represented in red color, and non-hot spots are represented in blue color. **a** BID experimental verification data. **b** Prediction results of our model. Hot spots predicted correctly are colored in red, while non-hot spots predicted correctly are colored in blue. The residues in yellow (E70 for our method) are non-hot spots wrongly predicted to be hot spots. **c** Prediction results of Hu’method. Hot spots predicted correctly are colored in red, and non-hot spots predicted correctly are colored in blue. The residues in yellow (G38, E70 and L153) are non-hot spots wrongly predicted to be hot spots

To show the results of models clearly, Pymol was used to visualize our model’s predictions for a protein complex [44]. First, protein complex (PDB ID: 1DVA) from BID was chosen, as shown in Fig. 9, which consists of chain H and chain X. Chain H is factor DES-GLA FACTOR VIIA, and chain X is PEPTIDE E-76 peptide [45]. Experimental results verified that there are three hot spots and twelve non-hot spots on the interface of the chain H and chain X. Second, we tested our model on the protein complex. For the E-76 peptide (chain X), our method can correctly predict three hot spots and eleven non-hot spots, only one non-hot spot was wrongly predicted. To fully show the power of our model, the predictive visualization results of Hu’s method have been investigated. For Hu’method, three non-hot spots were wrongly predicted, although all the hot spots were correctly predicted. In summary, our model perfoms good for predicting hot spot residues.

## Conclusion

This paper proposed a novel ensemble system that integrates feature selection and two types of base classifiers to achieve the best performance in hot spot prediction. It is worth mentioning that we only used the amino acid sequence information of protein and the feature of relative accessible surface area (relASA). Here, 46 descriptors of amino acids were obtained from AAindex1 database. Next, auto-correlation function was combined with the idea of sliding window to obtain amino acid features for protein sequence. Finally, the encoded data was respectively input into ensemble model containing SVM and KNN base classifiers. The model has been fully trained and tested, then the optimal ensemble model was obtained by means of majority voting. To sum up, the ensemble model with the top 83 classifiers yielded the best performance on training and test datasets. On the ASEdb and BID, the model achieved F1 scores of 0.92 and 0.89, respectively. Afterwards, based on different independent test sets (SKEMPI, dbMPIKT and Mix datasets), our model achieved good F1 scores of 0.8579, 0.8472 and 0.8657, respectively. In comparison with other the state-of-the-art methods, our model performs the best.

## Additional files

Additional file 1 All dataset materials source and data. **Table S1.** The 46 relative independently properties of AAindex1 **Table S2.** Training data-set respectively on ASEdb data-set **Table S3.** Test dataset derived from BID **Table S4.** Test dataset derived from SKEMPI **Table S5.** Test dataset derived from dbMPIKT **Table S6.** Test dataset derived from Mix data-set (DOCX 270 kb)

Additional file 2 All relevant MATLAB code. A simple Matlab implement of our predictor. (ZIP 19,742 kb)
