# Supplementary material for: Hot spot prediction in protein-protein interactions by an ensemble system
Source: BMC Syst Biol. 2018 Dec 31;12(Suppl 9):132. doi: 10.1186/s12918-018-0665-8 (PMC6311905; doi:10.1186/s12918-018-0665-8)
Supplement: Supplementary file 1 — All dataset materials source and data. Table S1. The 46 relative independently properties of AAindex1 Table S2. Training data-set respectively on ASEdb data-set Table S3. Test dataset derived from BID Table S4. Test dataset derived from SKEMPI Table S5. Test dataset derived from dbMPIKT Table S6. Test dataset derived from Mix data-set (DOCX 270 kb) [file 12918_2018_665_MOESM1_ESM.docx]

**Supplementary Materials**

**Table S1. The 46 relative independently properties of AAindex1**

| 1 | BUNA790103 | Spin-spin coupling constants 3JHalpha-NH (Bundi-Wuthrich, 1979) |
| --- | --- | --- |
| 2 | QIAN880139 | Weights for coil at the window position of 6 (Qian-Sejnowski, 1988) |
| 3 | TANS770108 | Normalized frequency of zeta R (Tanaka-Scheraga, 1977) |
| 4 | VASM830101 | Relative population of conformational state A (Vasquez et al., 1983) |
| 5 | WERD780103 | Free energy change of alpha(Ri) to alpha(Rh) (Wertz-Scheraga, 1978) |
| 6 | MAXF760103 | Normalized frequency of zeta R (Maxfield-Scheraga, 1976) |
| 7 | RICJ880117 | Relative preference value at C" (Richardson-Richardson, 1988) |
| 8 | ROBB760107 | Information measure for extended without H-bond (Robson-Suzuki, 1976) |
| 9 | ROSM880103 | Loss of Side chain hydropathy by helix formation (Roseman, 1988) |
| 10 | WERD780102 | Free energy change of epsilon(i) to epsilon(ex) (Wertz-Scheraga, 1978) |
| 11 | WILM950104 | Hydrophobicity coefficient in RP-HPLC, C18 with 0.1%TFA/2-PrOH/MeCN/H2O (Wilce et al. 1995) |
| 12 | KHAG800101 | The Kerr-constant increments (Khanarian-Moore, 1980) |
| 13 | PRAM820103 | Correlation coefficient in regression analysis (Prabhakaran-Ponnuswamy, 1982) |
| 14 | QIAN880117 | Weights for beta-sheet at the window position of -3 (Qian-Sejnowski, 1988) |
| 15 | RACS820107 | Average relative fractional occurrence in A0(i-1) (Rackovsky-Scheraga, 1982) |
| 16 | VELV850101 | Electron-ion interaction potential (Veljkovic et al., 1985) |
| 17 | QIAN880123 | Weights for beta-sheet at the window position of 3 (Qian-Sejnowski, 1988) |
| 18 | RICJ880114 | Relative preference value at C1 (Richardson-Richardson, 1988) |
| 19 | SNEP660104 | Principal component IV (Sneath, 1966) |
| 20 | GEIM800103 | Alpha-helix indices for beta-proteins (Geisow-Roberts, 1980) |
| 21 | ISOY800107 | Normalized relative frequency of double bend (Isogai et al., 1980) |
| 22 | QIAN880128 | Weights for coil at the window position of -5 (Qian-Sejnowski, 1988) |
| 23 | ROBB760111 | Information measure for C-terminal turn (Robson-Suzuki, 1976) |
| 24 | AURR980118 | Normalized positional residue frequency at helix termini C" (Aurora-Rose, 1998) |
| 25 | AURR980120 | Normalized positional residue frequency at helix termini C4'' (Aurora-Rose, 1998) |
| 26 | NADH010107 | Hydropathy scale based on self-information values in the two-state model (50% accessibility) (Naderi-Manesh et al., 2001) |
| 27 | ISOY800106 | Normalized relative frequency of helix end (Isogai et al., 1980) |
| 28 | JOND920102 | Relative mutability (Jones et al., 1992) |
| 29 | PRAM820101 | Intercept in regression analysis (Prabhakaran-Ponnuswamy, 1982) |
| 30 | RICJ880101 | Relative preference value at N" (Richardson-Richardson, 1988) |
| 31 | RICJ880104 | Relative preference value at N1 (Richardson-Richardson, 1988) |
| 32 | SUEM840102 | Zimm-Bragg parameter sigma x 1.0E4 (Sueki et al., 1984) |
| 33 | VASM830102 | Relative population of conformational state C (Vasquez et al., 1983) |
| 34 | GEOR030103 | Linker propensity from 2-linker dataset (George-Heringa, 2003) |
| 35 | CHAM830102 | A parameter defined from the residuals obtained from the best correlation of the Chou-Fasman parameter of beta-sheet (Charton-Charton, 1983) |
| 36 | NAKH900113 | Ratio of average and computed composition (Nakashima et al., 1990) |
| 37 | RICJ880105 | Relative preference value at N2 (Richardson-Richardson, 1988) |
| 38 | WOLS870103 | Principal property value z3 (Wold et al., 1987) |
| 39 | GEOR030107 | Linker propensity from long dataset (linker length is greater than 14 residues) (George-Heringa, 2003) |
| 40 | QIAN880113 | Weights for alpha-helix at the window position of 6 (Qian-Sejnowski, 1988) |
| 41 | QIAN880129 | Weights for coil at the window position of -4 (Qian-Sejnowski, 1988) |
| 42 | RACS820103 | Average relative fractional occurrence in AL(i) (Rackovsky-Scheraga, 1982) |
| 43 | RACS820104 | Average relative fractional occurrence in EL(i) (Rackovsky-Scheraga, 1982)' |
| 44 | FASG760104 | pK-N (Fasman, 1976) |
| 45 | JOND750102 | pK (-COOH) (Jones, 1975) |
| 46 | KLEP840101 | Net charge (Klein et al., 1984) |

**Table S2. Training data-set respectively on ASEdb data-set**

| **PDB_ID** | **Residue** | **Chain** | **ΔΔGobs** | **Observed** | **PDB_ID** | **Residue** | **Chain** | **ΔΔG_obs_** | **Observed** |
| --- | --- | --- | --- | --- | --- | --- | --- | --- | --- |
| 1A4Y | W261 | A | 0.1 | -- | 1DVF | Y98 | D | 4.7 | ** |
| 1A4Y | W263 | A | 1.2 | / | 1DVF | Q100 | D | 1.6 | / |
| 1A4Y | S289 | A | 0 | -- | 1DX5 | F34 | M | 2.6 | ** |
| 1A4Y | W318 | A | 1.5 | / | 1DX5 | Q38 | M | 1.4 | / |
| 1A4Y | K320 | A | -0.3 | -- | 1DX5 | R67 | M | 3.4 | ** |
| 1A4Y | E344 | A | 0.2 | -- | 1DX5 | T74 | M | 0.8 | / |
| 1A4Y | W375 | A | 1 | / | 1DX5 | R75 | M | 0.7 | / |
| 1A4Y | E401 | A | 0.9 | / | 1DX5 | Y76 | M | 3 | ** |
| 1A4Y | Y434 | A | 3.3 | ** | 1DX5 | K81 | M | 1 | / |
| 1A4Y | D435 | A | 3.5 | ** | 1DX5 | I82 | M | 2.6 | ** |
| 1A4Y | Y437 | A | 0.8 | / | 1DX5 | M84 | M | 0.3 | -- |
| 1A4Y | I459 | A | 0.7 | / | 1DX5 | K110 | M | 0 | -- |
| 1A4Y | R5 | B | 2.3 | ** | 1GC1 | Q25 | C | 0.03 | -- |
| 1A4Y | H8 | B | 0.9 | / | 1GC1 | H27 | C | 0.28 | -- |
| 1A4Y | Q12 | B | 0.3 | -- | 1GC1 | K29 | C | 0.59 | / |
| 1A4Y | R31 | B | 0.2 | -- | 1GC1 | N32 | C | 0.18 | -- |
| 1A4Y | R32 | B | 0.9 | / | 1GC1 | Q33 | C | 0.1 | -- |
| 1A4Y | N68 | B | 0.2 | -- | 1GC1 | K35 | C | 0.32 | -- |
| 1A4Y | H84 | B | 0.2 | -- | 1GC1 | Q40 | C | -0.41 | -- |
| 1A4Y | W89 | B | 0.2 | -- | 1GC1 | S42 | C | 0 | -- |
| 1A4Y | E108 | B | -0.3 | -- | 1GC1 | L44 | C | 1.04 | / |
| 1A4Y | H114 | B | 0.65 | / | 1GC1 | T45 | C | -0.15 | -- |
| 1AHW | Y156 | C | 4 | ** | 1GC1 | N52 | C | 0.7 | / |
| 1AHW | T167 | C | 0 | -- | 1GC1 | R59 | C | 1.16 | / |
| 1AHW | T170 | C | 1 | / | 1GC1 | S60 | C | -0.09 | -- |
| 1AHW | V198 | C | -0.3 | -- | 1GC1 | D63 | C | -0.32 | -- |
| 1BRS | K27 | A | 5.4 | ** | 1GC1 | Q64 | C | 0.44 | / |
| 1BRS | N58 | A | 3.1 | ** | 1JTG | E104 | A | 1.55 | / |
| 1BRS | R59 | A | 5.2 | ** | 1JTG | Y105 | A | -0.17 | -- |
| 1BRS | E60 | A | -0.2 | -- | 1JTG | S130 | A | 0.8 | / |
| 1BRS | R83 | A | 5.4 | ** | 1JTG | K234 | A | 1.4 | / |
| 1BRS | R87 | A | 5.5 | ** | 1JTG | S235 | A | 1.3 | / |
| 1BRS | H102 | A | 6.1 | ** | 1JTG | R243 | A | 1.4 | / |
| 1BRS | Y29 | D | 3.4 | ** | 1JTG | D49 | B | 1.8 | / |
| 1BRS | D35 | D | 4.5 | ** | 1JTG | K74 | B | 3.56 | ** |
| 1BRS | D39 | D | 7.7 | ** | 1JTG | F142 | B | 2.1 | ** |
| 1BRS | T42 | D | 1.8 | / | 1JTG | Y143 | B | 0.38 | -- |
| 1BRS | E76 | D | 1.3 | / | 1NMB | D56 | H | 2.8 | ** |
| 1BXI | C23 | A | 0.92 | / | 1NMB | Y99 | H | 1.5 | / |
| 1BXI | N24 | A | 0.14 | -- | 1NMB | Y100 | H | 0.5 | / |
| 1BXI | T27 | A | 0.73 | / | 1NMB | Y32 | L | 1.7 | / |
| 1BXI | S28 | A | 0.17 | -- | 1NMB | T93 | L | 0.3 | -- |
| 1BXI | S29 | A | 0.96 | / | 1NMB | L94 | L | 0.9 | / |
| 1BXI | E30 | A | 1.41 | / | 1VFB | W52 | B | 1.23 | / |
| 1BXI | L33 | A | 3.42 | ** | 1VFB | D58 | B | -0.2 | -- |
| 1BXI | V34 | A | 2.58 | ** | 1VFB | E98 | B | 1.1 | / |
| 1BXI | V37 | A | 1.66 | / | 1VFB | Y101 | B | 4 | ** |
| 1BXI | T38 | A | 0.9 | / | 3HFM | S31 | H | 0.2 | -- |
| 1BXI | E41 | A | 2.08 | ** | 3HFM | D32 | H | 2 | ** |
| 1BXI | S48 | A | 0.01 | -- | 3HFM | Y33 | H | 6 | ** |
| 1BXI | S50 | A | 2.19 | ** | 3HFM | Y50 | H | 7.5 | ** |
| 1BXI | D51 | A | 5.92 | ** | 3HFM | Y53 | H | 3.29 | ** |
| 1BXI | I53 | A | 0.85 | / | 3HFM | Y58 | H | 1.7 | / |
| 1BXI | Y54 | A | 4.83 | ** | 3HFM | N31 | L | 5.25 | ** |
| 1BXI | Y55 | A | 4.63 | ** | 3HFM | N32 | L | 5.2 | ** |
| 1CBW | T11 | D | 0.2 | -- | 3HFM | Y50 | L | 4.6 | ** |
| 1CBW | K15 | D | 2 | ** | 3HFM | Q53 | L | 1 | / |
| 1CBW | R17 | D | 0.5 | / | 3HFM | Y96 | L | 2.8 | ** |
| 1CBW | R39 | D | 0.2 | -- | 3HFM | H15 | Y | -0.44 | -- |
| 1DAN | L39 | L | 0 | -- | 3HFM | Y20 | Y | 5 | -- |
| 1DAN | K62 | L | 0 | -- | 3HFM | R21 | Y | 1 | / |
| 1DAN | Q64 | L | 0.8 | / | 3HFM | W63 | Y | 0.31 | -- |
| 1DAN | I69 | L | 1.9 | / | 3HFM | R73 | Y | -0.2 | -- |
| 1DAN | F71 | L | 1.2 | / | 3HFM | L75 | Y | 1.25 | / |
| 1DAN | L73 | L | 0 | -- | 3HFM | T89 | Y | 0 | -- |
| 1DAN | E77 | L | 0 | -- | 3HFM | N93 | Y | 0.6 | / |
| 1DAN | R79 | L | 1.2 | / | 3HFM | K96 | Y | 7 | ** |
| 1DAN | Q88 | L | 0 | -- | 3HFM | K97 | Y | 6 | ** |
| 1DAN | V92 | L | 0 | -- | 3HFM | I98 | Y | 0 | -- |
| 1DAN | N93 | L | 0 | -- | 3HFM | S100 | Y | 0.26 | -- |
| 1DAN | E94 | L | 0 | -- | 3HFM | D101 | Y | 0.94 | / |
| 1DAN | H115 | L | 0 | -- | 3HHR | I4 | A | 0.41 | / |
| 1DAN | T17 | T | 0.1 | -- | 3HHR | R8 | A | 0.2 | -- |
| 1DAN | K20 | T | 2.6 | ** | 3HHR | L9 | A | -0.04 | -- |
| 1DAN | I22 | T | 0.7 | / | 3HHR | N12 | A | 0.1 | -- |
| 1DAN | E24 | T | 0.7 | / | 3HHR | L15 | A | 0.15 | -- |
| 1DAN | Q37 | T | 0.55 | / | 3HHR | R16 | A | 0.24 | -- |
| 1DAN | K41 | T | 0.35 | -- | 3HHR | H18 | A | -0.5 | -- |
| 1DAN | S42 | T | -0.1 | -- | 3HHR | H21 | A | 0.2 | -- |
| 1DAN | D44 | T | 0.7 | / | 3HHR | Q22 | A | -0.2 | -- |
| 1DAN | W45 | T | 1.6 | / | 3HHR | F25 | A | -0.4 | -- |
| 1DAN | S47 | T | 0.05 | -- | 3HHR | Y42 | A | 0.2 | -- |
| 1DAN | K48 | T | 0.4 | / | 3HHR | L45 | A | 1.2 | / |
| 1DAN | F50 | T | 0.4 | / | 3HHR | Q46 | A | 0.1 | -- |
| 1DAN | D58 | T | 2.18 | ** | 3HHR | S62 | A | 0.2 | -- |
| 1DAN | D61 | T | 0.24 | -- | 3HHR | N63 | A | 0.3 | -- |
| 1DAN | F76 | T | 1.2 | / | 3HHR | R64 | A | 1.6 | / |
| 1DAN | Y94 | U | 1 | / | 3HHR | Q68 | A | 0.6 | / |
| 1DAN | Q110 | U | 1.4 | / | 3HHR | Y164 | A | 0.3 | -- |
| 1DAN | E128 | U | 0.1 | -- | 3HHR | R167 | A | 0.3 | -- |
| 1DAN | R131 | U | 0 | -- | 3HHR | K168 | A | -0.2 | -- |
| 1DAN | T132 | U | 0 | -- | 3HHR | D171 | A | 0.8 | / |
| 1DAN | L133 | U | 0 | -- | 3HHR | K172 | A | 2 | / |
| 1DAN | R135 | U | 0.55 | / | 3HHR | E174 | A | -0.9 | -- |
| 1DAN | F140 | U | 1.5 | / | 3HHR | T175 | A | 2 | ** |
| 1DAN | S163 | U | 0 | -- | 3HHR | R178 | A | 2.4 | ** |
| 1DAN | T203 | U | 0.1 | -- | 3HHR | I179 | A | 0.8 | / |
| 1DAN | V207 | U | -0.2 | -- | 3HHR | C182 | A | 1.01 | / |
| 1DAN | E208 | U | 0 | -- | 3HHR | R43 | B | 2.2 | ** |
| 1DVF | H30 | A | 1.7 | / | 3HHR | E44 | B | 1.8 | / |
| 1DVF | Y32 | A | 2 | ** | 3HHR | W76 | B | 0.6 | / |
| 1DVF | Y49 | A | 1.7 | / | 3HHR | T77 | B | -0.25 | -- |
| 1DVF | Y50 | A | 0.7 | / | 3HHR | S102 | B | -0.2 | -- |
| 1DVF | W92 | A | 0.3 | -- | 3HHR | I103 | B | 1.8 | / |
| 1DVF | T30 | B | 0.9 | / | 3HHR | W104 | B | 4.5 | ** |
| 1DVF | Y32 | B | 1.8 | / | 3HHR | I105 | B | 2 | ** |
| 1DVF | W52 | B | 4.2 | ** | 3HHR | C108 | B | 0 | -- |
| 1DVF | D54 | B | 4.3 | ** | 3HHR | E120 | B | -0.2 | -- |
| 1DVF | N56 | B | 1.2 | / | 3HHR | K121 | B | 0.1 | -- |
| 1DVF | D58 | B | 1.6 | / | 3HHR | C122 | B | 0 | -- |
| 1DVF | E98 | B | 4.2 | ** | 3HHR | D126 | B | 1 | / |
| 1DVF | R99 | B | 1.9 | / | 3HHR | E127 | B | 1 | / |
| 1DVF | D100 | B | 2.8 | ** | 3HHR | D164 | B | 1.6 | / |
| 1DVF | Y101 | B | 4 | ** | 3HHR | I165 | B | 2.2 | ** |
| 1DVF | K30 | D | 0.9 | / | 3HHR | Q166 | B | 0 | -- |
| 1DVF | H33 | D | 1.9 | / | 3HHR | K167 | B | 0 | -- |
| 1DVF | I97 | D | 2.7 | ** | 3HHR | W169 | B | 4.5 | ** |
| 3HHR | N218 | B | 0.3 | -- | 3HHR | R217 | B | 0.2 | -- |

** represents hot-spots

-- represents non-hot spots

/ represents the residue not included

**3. Table S3^&^ Test dataset derived from BID**

| **PDB_ID** | **Residue** | **Chain** | **Strength^1^** | **Observed** | **PDB_ID** | **Residue** | **Chain** | **Strength^1^** | **Observed** |
| --- | --- | --- | --- | --- | --- | --- | --- | --- | --- |
| 1CDL | F12 | A | N | -- | 2NMB | M99 | A | S | ** |
| 1CDL | F19 | A | W | -- | 2NMB | C150 | A | S | ** |
| 1CDL | F92 | A | S | ** | 2NMB | Y2 | B | I | -- |
| 1CDL | K799 | E | N | -- | 2NMB | I3 | B | I | -- |
| 1CDL | W800 | E | S | ** | 3SAK | E8 | A | W | -- |
| 1CDL | K802 | E | I | -- | 3SAK | F10 | A | S | ** |
| 1CDL | R808 | E | I | -- | 3SAK | T11 | A | I | -- |
| 1CDL | I810 | E | S | ** | 3SAK | L12 | A | S | ** |
| 1CDL | L813 | E | S | ** | 3SAK | Q13 | A | W | -- |
| 1DDM | I144 | A | S | ** | 3SAK | I14 | A | S | ** |
| 1DDM | E145 | A | S | ** | 3SAK | R15 | A | I | -- |
| 1DDM | K146 | A | I | -- | 3SAK | R17 | A | W | -- |
| 1DDM | S148 | A | N | -- | 3SAK | R19 | A | S | ** |
| 1DDM | C150 | A | S | ** | 3SAK | F20 | A | S | ** |
| 1DDM | R165 | A | I | -- | 3SAK | F23 | A | S | ** |
| 1DDM | C198 | A | S | ** | 3SAK | L26 | A | S | ** |
| 1DDM | F2 | B | I | -- | 3SAK | N27 | A | I | -- |
| 1DDM | S3 | B | S | ** | 3SAK | L30 | A | S | ** |
| 1DDM | N4 | B | S | ** | 3SAK | D34 | A | W | -- |
| 1DDM | M5 | B | S | ** | 1CDL | G804 | E | S | ** |
| 1DDM | S6 | B | S | ** | 1CDL | G811 | E | I | -- |
| 1DDM | F7 | B | S | ** | 1CDL | R812 | E | S | ** |
| 1DDM | E8 | B | W | -- | 1DVA | G38 | H | I | -- |
| 1DDM | F10 | B | W | -- | 1DVA | A1 | X | N | -- |
| 1DFJ | K7 | E | S | ** | 1DVA | D5 | X | W | -- |
| 1DVA | L32 | H | S | ** | 1DVA | Q14 | X | N | -- |
| 1DVA | L34 | H | S | ** | 1DVA | V16 | X | N | -- |
| 1DVA | N37 | H | N | -- | 1DX5 | I24 | N | N | -- |
| 1DVA | I65 | H | N | -- | 1DX5 | F34 | N | I | -- |
| 1DVA | V67 | H | N | -- | 1DX5 | K36 | N | W | -- |
| 1DVA | E70 | H | W | -- | 1DX5 | P37 | N | W | -- |
| 1DVA | L73 | H | N | -- | 1DX5 | Q38 | N | W | -- |
| 1DVA | S74 | H | N | -- | 1DX5 | E39 | N | N | -- |
| 1DVA | E75 | H | N | -- | 1DX5 | L65 | N | W | -- |
| 1DVA | H76 | H | S | ** | 1DX5 | R67 | N | S | ** |
| 1DVA | E80 | H | N | -- | 1DX5 | E80 | N | S | ** |
| 1DVA | S82 | H | N | -- | 1DX5 | K81 | N | W | -- |
| 1DVA | L144 | H | N | -- | 1DX5 | I82 | N | I | -- |
| 1DVA | L153 | H | W | -- | 1DX5 | M84 | N | N | -- |
| 1DVA | L2 | X | S | ** | 1DX5 | K110 | N | N | -- |
| 1DVA | R7 | X | W | -- | 1DX5 | K235 | N | N | -- |
| 1DVA | V8 | X | I | -- | 1EBP | G9 | C | I | -- |
| 1DVA | D9 | X | I | -- | 1EBP | P10 | **C** | I | -- |
| 1DVA | W11 | X | S | ** | 1ES7 | V26 | A | I | -- |
| 1DVA | Y12 | X | S | ** | 1ES7 | W31 | A | S | ** |
| 1DVA | F15 | X | S | ** | 1ES7 | P50 | A | I | -- |
| 1DZI | N154 | A | I | -- | 1FAK | Q37 | T | W | -- |
| 1DZI | Y157 | A | S | ** | 1FAK | K41 | T | N | -- |
| 1DZI | Q215 | A | S | ** | 1FAK | S42 | T | N | -- |
| 1DZI | D219 | A | I | -- | 1FAK | D44 | T | W | -- |
| 1DZI | L220 | A | N | -- | 1FAK | Y94 | T | W | -- |
| 1DZI | T221 | A | S | ** | 1FAK | K15 | T | N | -- |
| 1DZI | E256 | A | N | -- | 1FAK | T17 | T | N | -- |
| 1DZI | H258 | A | I | -- | 1FAK | N18 | T | N | -- |
| 1EBP | F93 | A | S | ** | 1FAK | K20 | T | S | ** |
| 1EBP | M150 | A | S | ** | 1FAK | I22 | T | W | -- |
| 1EBP | T151 | A | W | -- | 1FAK | E24 | T | W | -- |
| 1EBP | F205 | A | S | ** | 1FAK | S47 | T | N | -- |
| 1EBP | L11 | C | N | -- | 1FAK | S48 | T | N | -- |
| 1EBP | T12 | C | I | -- | 1FAK | F50 | T | N | -- |
| 1EBP | W13 | C | S | ** | 1FAK | D58 | T | S | ** |
| 1ES7 | F49 | A | I | -- | 1FAK | E128 | T | N | -- |
| 1FCC | E27 | C | S | ** | 1FAK | L133 | T | N | -- |
| 1FCC | K28 | C | W | -- | 1FAK | R135 | T | N | -- |
| 1FCC | K31 | C | S | ** | 1FAK | F140 | T | I | -- |
| 1FCC | N35 | C | I | -- | 1FAK | T203 | T | N | -- |
| 1FCC | D40 | C | N | -- | 1FAK | V207 | T | N | -- |
| 1FCC | E42 | C | N | -- | 1FE8 | R963 | A | N | -- |
| 1FCC | W43 | C | S | ** | 1FE8 | E987 | A | N | -- |
| 1FOE | S41 | B | I | -- | 1FE8 | H990 | A | N | -- |
| 1GL4 | R403 | A | I | -- | 1FE8 | H1023 | A | N | -- |
| 1GL4 | D427 | A | S | ** | 1FOE | G54 | B | S | ** |
| 1GL4 | H429 | A | S | ** | 1G3I | D438 | A | S | ** |
| 1GL4 | Y431 | A | S | ** | 1G3I | L439 | A | S | ** |
| 1GL4 | Y440 | A | I | -- | 1G3I | R441 | A | S | ** |
| 1GL4 | E616 | A | S | ** | 1G3I | F442 | A | S | ** |
| 1GL4 | R620 | A | S | ** | 1G3I | I443 | A | S | ** |
| 1JAT | E55 | A | S | ** | 1G3I | L444 | A | S | ** |
| 1JAT | F8 | B | S | ** | 1IHB | N101 | B | N | -- |
| 1K4U | R368 | P | S | ** | 1IHB | R133 | B | W | -- |
| 1K4U | L373 | P | W | -- | 1IHB | H135 | B | W | -- |
| 1K4U | I374 | P | S | ** | 1IHB | K136 | B | I | -- |
| 1K4U | R377 | P | W | -- | 1JPP | K345 | B | S | ** |
| 1K4U | T382 | P | I | -- | 1JPP | K354 | B | N | -- |
| 1LQB | M561 | D | N | -- | 1JPP | W383 | B | S | ** |
| 1LQB | L562 | D | N | -- | 1JPP | R386 | B | I | -- |
| 1MQ8 | T206 | B | S | ** | 1JPP | K435 | B | I | -- |
| 1NFI | Y181 | F | S | ** | 1JPP | R469 | B | I | -- |
| 1NFI | C215 | F | N | -- | 1JPP | H470 | B | I | -- |
| 1UB4 | F453 | C | N | -- | 1NUN | D76 | A | I | -- |
| 1UB4 | L455 | C | S | ** | 1NUN | R78 | A | I | -- |
| 1UB4 | L458 | C | S | ** | 1NUN | R155 | A | I | -- |
| 2HHB | Y35 | D | N | -- |  |  |  |  |  |

^&^This table is derived from Tuncbag *et.al ,Bioinformatics* (2009) and Zhu X *et.al, Proteins Structure Function & Bioinformatics*.

^1^Interaction strength; W (weak), I (intermediate), N (insignificant), S (strong).

** represents hot spots

-- represents non-hot spots

**4. Table S4^&^ Test dataset derived from SKEMPI**

| **PDB_ID** | **Residue** | **Chain** | **ΔΔG_com_** | **Observed** | **PDB_ID** | **Residue** | **Chain** | **ΔΔG_com_** | **Observed** |
| --- | --- | --- | --- | --- | --- | --- | --- | --- | --- |
| 1A22 | 3T | A | 0.050 | -- | 1EMV | 84S | B | 0.109 | -- |
| 1A22 | 7S | A | 0.340 | -- | 1EMV | 86F | B | 6.044 | ** |
| 1A22 | 8R | A | 0.200 | -- | 1EMV | 87T | B | 0.158 | -- |
| 1A22 | 9L | A | 0.040 | -- | 1EMV | 92Q | B | 0.278 | -- |
| 1A22 | 12N | A | 0.100 | -- | 1F47 | 8I | A | 2.515 | ** |
| 1A22 | 14M | A | 0.000 | -- | 1F47 | 9P | A | 0.058 | -- |
| 1A22 | 15L | A | 0.150 | -- | 1F47 | 11F | A | 2.444 | ** |
| 1A22 | 16R | A | 0.238 | -- | 1F47 | 12L | A | 2.294 | ** |
| 1A22 | 19R | A | 0.051 | -- | 1F47 | 14K | A | 0.043 | -- |
| 1A22 | 21H | A | 0.155 | -- | 1F47 | 15Q | A | 0.046 | -- |
| 1A22 | 22Q | A | 0.220 | -- | 1FC2 | 26F | C | 0.014 | -- |
| 1A22 | 25F | A | 0.229 | -- | 1FC2 | 27I | C | 3.729 | ** |
| 1A22 | 26D | A | 0.211 | -- | 1FCC | 25T | C | 0.240 | -- |
| 1A22 | 42Y | A | 0.221 | -- | 1FCC | 31K | C | 3.476 | ** |
| 1A22 | 46Q | A | 0.191 | -- | 1FCC | 35N | C | 2.364 | ** |
| 1A22 | 51S | A | 0.348 | -- | 1FCC | 40D | C | 0.272 | -- |
| 1A22 | 55S | A | 0.110 | -- | 1FCC | 42E | C | 0.385 | -- |
| 1A22 | 57S | A | 0.200 | -- | 1FCC | 43W | C | 3.771 | ** |
| 1A22 | 59P | A | 0.380 | -- | 1FFW | 23H | B | 0.034 | -- |
| 1A22 | 62S | A | 0.155 | -- | 1FFW | 44D | B | 0.074 | -- |
| 1A22 | 63N | A | 0.314 | -- | 1FFW | 49D | B | 0.096 | -- |
| 1A22 | 69Q | A | 0.050 | -- | 1FFW | 55C | B | 0.204 | -- |
| 1A22 | 73L | A | 0.200 | -- | 1FFW | 56F | B | 3.644 | ** |
| 1A22 | 153Y | A | 0.348 | -- | 1GC1 | 1K | C | 0.062 | -- |
| 1A22 | 156R | A | 0.278 | -- | 1GC1 | 2K | C | 0.017 | -- |
| 1A22 | 157K | A | 0.155 | -- | 1GC1 | 8K | C | 0.105 | -- |
| 1A22 | 161K | A | 2.014 | ** | 1GC1 | 10D | C | 0.000 | -- |
| 1A22 | 167R | A | 2.424 | ** | 1GC1 | 11T | C | 0.000 | -- |
| 1A22 | 169V | A | 0.000 | -- | 1GC1 | 15T | C | 0.322 | -- |
| 1A22 | 170Q | A | 0.270 | -- | 1GC1 | 17T | C | 0.128 | -- |
| 1A22 | 173S | A | 0.050 | -- | 1GC1 | 19S | C | 0.000 | -- |
| 1A22 | 175E | A | 0.012 | -- | 1GC1 | 20Q | C | 0.017 | -- |
| 1A22 | 176G | A | 0.340 | -- | 1GC1 | 21K | C | 0.128 | -- |
| 1A22 | 177S | A | 0.200 | -- | 1GC1 | 22K | C | 0.240 | -- |
| 1A22 | 180F | A | 0.191 | -- | 1GC1 | 23S | C | 0.293 | -- |
| 1A22 | 2K | B | 0.240 | -- | 1GC1 | 25Q | C | 0.032 | -- |
| 1A22 | 5K | B | 0.240 | -- | 1GC1 | 27H | C | 0.282 | -- |
| 1A22 | 7R | B | 0.269 | -- | 1GC1 | 30N | C | 0.170 | -- |
| 1A22 | 9P | B | 0.260 | -- | 1GC1 | 31S | C | 0.105 | -- |
| 1A22 | 15S | B | 0.015 | -- | 1GC1 | 32N | C | 0.183 | -- |
| 1A22 | 19T | B | 0.070 | -- | 1GC1 | 33Q | C | 0.105 | -- |
| 1A22 | 31N | B | 0.011 | -- | 1GC1 | 35K | C | 0.322 | -- |
| 1A22 | 32T | B | 0.077 | -- | 1GC1 | 42S | C | 0.000 | -- |
| 1A22 | 33Q | B | 0.194 | -- | 1GC1 | 45T | C | 0.149 | -- |
| 1A22 | 34E | B | 0.172 | -- | 1GC1 | 50K | C | 0.047 | -- |
| 1A22 | 36T | B | 0.025 | -- | 1GC1 | 53D | C | 0.302 | -- |
| 1A22 | 38E | B | 0.079 | -- | 1GC1 | 56D | C | 0.070 | -- |
| 1A22 | 39W | B | 0.050 | -- | 1GC1 | 58R | C | 0.132 | -- |
| 1A22 | 40K | B | 0.144 | -- | 1GC1 | 60S | C | 0.089 | -- |
| 1A22 | 50E | B | 0.178 | -- | 1GC1 | 66N | C | 0.034 | -- |
| 1A22 | 54Y | B | 0.199 | -- | 1GC1 | 72K | C | 0.017 | -- |
| 1A22 | 56N | B | 0.255 | -- | 1GC1 | 73N | C | 0.108 | -- |
| 1A22 | 57S | B | 0.161 | -- | 1GC1 | 75K | C | 0.158 | -- |
| 1A22 | 59F | B | 0.015 | -- | 1GC1 | 86V | C | 0.070 | -- |
| 1A22 | 61S | B | 0.028 | -- | 1GC1 | 87E | C | 0.218 | -- |
| 1A22 | 65P | B | 2.965 | ** | 1GC1 | 88D | C | 0.070 | -- |
| 1A22 | 67C | B | 0.000 | -- | 1GC1 | 89Q | C | 0.170 | -- |
| 1A22 | 69K | B | 0.043 | -- | 1GC1 | 90K | C | 0.047 | -- |
| 1A22 | 78D | B | 0.210 | -- | 1GC1 | 91E | C | 0.128 | -- |
| 1A22 | 79E | B | 0.057 | -- | 1GC1 | 92E | C | 0.016 | -- |
| 1A22 | 80K | B | 0.130 | -- | 1GC1 | 94Q | C | 0.108 | -- |
| 1A22 | 81C | B | 0.000 | -- | 1GCQ | 18P | C | 0.121 | -- |
| 1A22 | 83S | B | 0.237 | -- | 1GCQ | 19P | C | 0.085 | -- |
| 1A22 | 88V | B | 0.046 | -- | 1H9D | 94N | B | 2.110 | ** |
| 1A22 | 89Q | B | 0.030 | -- | 1IAR | 6T | A | 0.104 | -- |
| 1A22 | 107D | B | 0.314 | -- | 1IAR | 8Q | A | 0.022 | -- |
| 1A22 | 111R | B | 0.314 | -- | 1IAR | 11I | A | 0.069 | -- |
| 1A22 | 113E | B | 0.314 | -- | 1IAR | 15N | A | 0.034 | -- |
| 1A22 | 116R | B | 0.062 | -- | 1IAR | 16S | A | 0.183 | -- |
| 1A22 | 120I | B | 2.164 | ** | 1IAR | 19E | A | 0.320 | -- |
| 1A22 | 121Q | B | 0.009 | -- | 1IAR | 77K | A | 0.154 | -- |
| 1A22 | 122K | B | 0.027 | -- | 1IAR | 78Q | A | 0.125 | -- |
| 1A22 | 128E | B | 0.083 | -- | 1IAR | 82F | A | 0.086 | -- |
| 1A22 | 130E | B | 0.079 | -- | 1IAR | 84K | A | 0.345 | -- |
| 1A22 | 134K | B | 0.000 | -- | 1IAR | 88R | A | 3.753 | ** |
| 1A22 | 135E | B | 0.199 | -- | 1JCK | 90Y | B | 2.595 | ** |
| 1A22 | 138E | B | 0.120 | -- | 1JCK | 91V | B | 2.232 | ** |
| 1A22 | 140K | B | 0.120 | -- | 1JCK | 176F | B | 2.133 | ** |
| 1A22 | 142K | B | 0.120 | -- | 1JRH | 37K | I | 3.714 | ** |
| 1A22 | 145D | B | 0.120 | -- | 1JRH | 38N | I | 0.170 | -- |
| 1A22 | 149T | B | 0.202 | -- | 1JRH | 39Y | I | 3.527 | ** |
| 1A22 | 150T | B | 0.097 | -- | 1JRH | 40G | I | 4.450 | ** |
| 1A22 | 166R | B | 0.056 | -- | 1JRH | 42K | I | 3.386 | ** |
| 1A22 | 168R | B | 0.190 | -- | 1JRH | 43N | I | 4.299 | ** |
| 1A22 | 172R | B | 0.268 | -- | 1JRH | 44S | I | 0.378 | -- |
| 1A22 | 173N | B | 0.298 | -- | 1JRH | 69N | I | 0.200 | -- |
| 1A22 | 174S | B | 0.034 | -- | 1JRH | 71L | I | 2.959 | ** |
| 1A22 | 179E | B | 0.108 | -- | 1JRH | 72W | I | 4.429 | ** |
| 1A4Y | 5R | B | 6.936 | ** | 1JRH | 73V | I | 0.214 | -- |
| 1A4Y | 12Q | B | 0.300 | -- | 1JRH | 74R | I | 0.393 | -- |
| 1A4Y | 13H | B | 0.297 | -- | 1JRH | 88K | I | 0.312 | -- |
| 1A4Y | 31R | B | 0.250 | -- | 1JRH | 92W | L | 2.818 | ** |
| 1A4Y | 33R | B | 0.327 | -- | 1JRH | 94T | L | 0.385 | -- |
| 1A4Y | 66R | B | 0.203 | -- | 1JRH | 54W | H | 2.686 | ** |
| 1A4Y | 68N | B | 0.118 | -- | 1JRH | 55W | H | 2.421 | ** |
| 1A4Y | 70R | B | 0.232 | -- | 1JRH | 103F | H | 0.000 | -- |
| 1A4Y | 84H | B | 0.170 | -- | 1JTG | 36F | B | 3.415 | ** |
| 1A4Y | 89W | B | 0.240 | -- | 1JTG | 41H | B | 2.950 | ** |
| 1A4Y | 108E | B | 0.323 | -- | 1JTG | 53Y | B | 2.690 | ** |
| 1A4Y | 261W | A | 0.101 | -- | 1JTG | 71S | B | 2.520 | ** |
| 1A4Y | 287E | A | 0.101 | -- | 1JTG | 74K | B | 3.906 | ** |
| 1A4Y | 289S | A | 0.042 | -- | 1JTG | 112W | B | 3.033 | ** |
| 1A4Y | 320K | A | 0.310 | -- | 1JTG | 113S | B | 2.286 | ** |
| 1A4Y | 344E | A | 0.179 | -- | 1JTG | 142F | B | 3.546 | ** |
| 1A4Y | 430Q | A | 0.072 | -- | 1JTG | 143Y | B | 3.138 | ** |
| 1A4Y | 432V | A | 0.072 | -- | 1JTG | 148H | B | 2.683 | ** |
| 1A4Y | 434Y | A | 7.207 | ** | 1JTG | 150W | B | 4.132 | ** |
| 1A4Y | 435D | A | 6.248 | ** | 1JTG | 160R | B | 2.631 | ** |
| 1A4Y | 437Y | A | 6.315 | ** | 1JTG | 162W | B | 2.727 | ** |
| 1A4Y | 457R | A | 0.224 | -- | 1JTG | 74Q | A | 2.972 | ** |
| 1AHW | 156T | C | 0.074 | -- | 1JTG | 75N | A | 2.142 | ** |
| 1AHW | 187V | C | 0.314 | -- | 1JTG | 78V | A | 3.750 | ** |
| 1AK4 | 85P | D | 2.449 | ** | 1JTG | 79E | A | 2.080 | ** |
| 1AK4 | 86V | D | 2.355 | ** | 1JTG | 80Y | A | 2.276 | ** |
| 1AK4 | 87H | D | 2.373 | ** | 1JTG | 85E | A | 4.529 | ** |
| 1AK4 | 89G | D | 3.440 | ** | 1JTG | 104M | A | 2.001 | ** |
| 1AK4 | 90P | D | 3.535 | ** | 1JTG | 105S | A | 2.039 | ** |
| 1AK4 | 93P | D | 2.046 | ** | 1JTG | 143E | A | 2.421 | ** |
| 1BRS | 25K | A | 6.484 | ** | 1JTG | 190V | A | 0.302 | -- |
| 1BRS | 56N | A | 3.089 | ** | 1JTG | 208K | A | 2.648 | ** |
| 1BRS | 57R | A | 6.526 | ** | 1JTG | 217R | A | 2.459 | ** |
| 1BRS | 58E | A | 0.094 | -- | 1KTZ | 64R | A | 2.883 | ** |
| 1BRS | 71E | A | 4.135 | ** | 1KTZ | 3L | B | 2.270 | ** |
| 1BRS | 85R | A | 6.723 | ** | 1KTZ | 6F | B | 3.425 | ** |
| 1BRS | 100H | A | 6.935 | ** | 1KTZ | 26I | B | 2.342 | ** |
| 1BRS | 29Y | D | 7.141 | ** | 1LFD | 35K | A | 0.258 | -- |
| 1BRS | 35D | D | 6.575 | ** | 1LFD | 43D | A | 0.280 | -- |
| 1BRS | 39D | D | 7.666 | ** | 1LFD | 44E | A | 0.246 | -- |
| 1BRS | 42T | D | 5.941 | ** | 1MAH | 327Y | A | 0.386 | -- |
| 1BRS | 74E | D | 4.673 | ** | 1NMB | 103Y | H | 2.140 | ** |
| 1BRS | 78E | D | 4.327 | ** | 1PPF | 14P | I | 0.122 | -- |
| 1CBW | 11T | I | 0.220 | -- | 1PPF | 17T | I | 3.145 | ** |
| 1CBW | 13P | I | 0.056 | -- | 1PPF | 18L | I | 3.541 | ** |
| 1CBW | 15K | I | 2.091 | ** | 1PPF | 20Y | I | 3.166 | ** |
| 1CBW | 19I | I | 0.141 | -- | 1PPF | 21R | I | 0.205 | -- |
| 1CBW | 20R | I | 0.350 | -- | 1PPF | 42V | I | 2.581 | ** |
| 1CBW | 33F | I | 0.141 | -- | 1R0R | 13L | I | 3.775 | ** |
| 1CBW | 34V | I | 0.051 | -- | 1R0R | 14E | I | 2.060 | ** |
| 1CBW | 39R | I | 0.220 | -- | 1R0R | 15Y | I | 5.398 | ** |
| 1CBW | 46K | I | 0.141 | -- | 1R0R | 16R | I | 0.095 | -- |
| 1CHO | 10K | I | 0.179 | -- | 1R0R | 27G | I | 3.081 | ** |
| 1CHO | 11P | I | 0.375 | -- | 1R0R | 31N | I | 0.033 | -- |
| 1CHO | 14T | I | 4.179 | ** | 1REW | 38F | A | 2.123 | ** |
| 1CHO | 15L | I | 6.271 | ** | 1REW | 39P | A | 2.088 | ** |
| 1CHO | 16E | I | 2.301 | ** | 1REW | 42D | A | 0.065 | -- |
| 1CHO | 17Y | I | 2.508 | ** | 1REW | 77S | A | 0.193 | -- |
| 1CHO | 18R | I | 3.147 | ** | 1REW | 89L | A | 0.176 | -- |
| 1CHO | 39V | I | 5.095 | ** | 1REW | 38F | B | 2.123 | ** |
| 1DAN | 10K | T | 0.397 | -- | 1REW | 39P | B | 2.088 | ** |
| 1DAN | 11S | T | 0.130 | -- | 1REW | 42D | B | 0.065 | -- |
| 1DAN | 12T | T | 0.120 | -- | 1REW | 77S | B | 0.193 | -- |
| 1DAN | 13N | T | 0.180 | -- | 1REW | 89L | B | 0.176 | -- |
| 1DAN | 15K | T | 3.664 | ** | 1REW | 55Q | C | 2.657 | ** |
| 1DAN | 16T | T | 0.159 | -- | 1S1Q | 42F | A | 0.196 | -- |
| 1DAN | 21E | T | 0.101 | -- | 1S1Q | 73W | A | 0.276 | -- |
| 1DAN | 23K | T | 0.117 | -- | 1TM1 | 39T | I | 2.896 | ** |
| 1DAN | 28V | T | 0.193 | -- | 1TM1 | 41E | I | 2.530 | ** |
| 1DAN | 31V | T | 0.132 | -- | 1TM1 | 42Y | I | 2.579 | ** |
| 1DAN | 33I | T | 0.132 | -- | 1TM1 | 46R | I | 3.960 | ** |
| 1DAN | 37S | T | 0.069 | -- | 1TM1 | 48R | I | 3.689 | ** |
| 1DAN | 38G | T | 0.067 | -- | 1TM1 | 51V | I | 0.025 | -- |
| 1DAN | 39D | T | 2.945 | ** | 1VFB | 92W | A | 3.357 | ** |
| 1DAN | 42S | T | 0.044 | -- | 1VFB | 93S | A | 0.343 | -- |
| 1DAN | 46Y | T | 0.132 | -- | 1VFB | 30T | B | 0.056 | -- |
| 1DAN | 53D | T | 3.000 | ** | 1VFB | 56N | B | 0.178 | -- |
| 1DAN | 54L | T | 0.000 | -- | 1VFB | 58D | B | 0.207 | -- |
| 1DAN | 55T | T | 3.577 | ** | 1VFB | 99R | B | 0.100 | -- |
| 1DAN | 56D | T | 0.273 | -- | 1VFB | 100D | B | 3.126 | ** |
| 1DAN | 57E | T | 0.152 | -- | 1VFB | 19N | C | 0.396 | -- |
| 1DAN | 58I | T | 0.000 | -- | 1VFB | 24S | C | 2.110 | ** |
| 1DAN | 59V | T | 0.000 | -- | 1VFB | 121Q | C | 2.660 | ** |
| 1DAN | 63K | T | 0.138 | -- | 1VFB | 124I | C | 2.662 | ** |
| 1DAN | 64Q | T | 0.103 | -- | 1VFB | 125R | C | 2.615 | ** |
| 1DAN | 67L | T | 0.060 | -- | 1XD3 | 8L | B | 2.738 | ** |
| 1DAN | 2P | U | 0.193 | -- | 1XD3 | 24E | B | 0.246 | -- |
| 1DAN | 9E | U | 0.175 | -- | 1XD3 | 27K | B | 0.064 | -- |
| 1DAN | 15E | U | 0.261 | -- | 1XD3 | 44I | B | 0.272 | -- |
| 1DAN | 16T | U | 0.060 | -- | 1XD3 | 52D | B | 0.246 | -- |
| 1DAN | 17N | U | 0.207 | -- | 1XD3 | 58D | B | 0.246 | -- |
| 1DAN | 24Q | U | 0.034 | -- | 1Z7X | 263W | W | 2.211 | ** |
| 1DAN | 27E | U | 0.034 | -- | 1Z7X | 434Y | W | 6.786 | ** |
| 1DAN | 32K | U | 0.121 | -- | 1Z7X | 435D | W | 5.414 | ** |
| 1DAN | 38E | U | 0.086 | -- | 1Z7X | 437Y | W | 4.930 | ** |
| 1DAN | 39D | U | 0.027 | -- | 1Z7X | 459I | W | 0.337 | -- |
| 1DAN | 40E | U | 0.022 | -- | 2FTL | 12G | I | 4.346 | ** |
| 1DAN | 41R | U | 0.000 | -- | 2FTL | 15K | I | 10.289 | ** |
| 1DAN | 42T | U | 0.000 | -- | 2FTL | 18I | I | 4.969 | ** |
| 1DAN | 43L | U | 0.028 | -- | 2FTL | 36G | I | 2.191 | ** |
| 1DAN | 46R | U | 0.073 | -- | 2G2U | 36F | B | 2.762 | ** |
| 1DAN | 47N | U | 0.206 | -- | 2G2U | 50Y | B | 2.200 | ** |
| 1DAN | 48N | U | 0.103 | -- | 2G2U | 53Y | B | 2.301 | ** |
| 1DAN | 49T | U | 0.017 | -- | 2G2U | 73E | B | 2.088 | ** |
| 1DAN | 50F | U | 3.451 | ** | 2G2U | 74K | B | 0.217 | -- |
| 1DAN | 54R | U | 0.003 | -- | 2G2U | 112W | B | 0.274 | -- |
| 1DAN | 55D | U | 0.011 | -- | 2G2U | 142F | B | 0.276 | -- |
| 1DAN | 56V | U | 0.199 | -- | 2G2U | 144R | B | 0.342 | -- |
| 1DAN | 57F | U | 0.060 | -- | 2I9B | 129R | E | 0.287 | -- |
| 1DAN | 59K | U | 0.101 | -- | 2I9B | 134R | E | 0.363 | -- |
| 1DAN | 60D | U | 0.101 | -- | 2J0T | 2T | D | 4.387 | ** |
| 1DAN | 62I | U | 0.179 | -- | 2J0T | 68S | D | 3.694 | ** |
| 1DAN | 67Y | U | 0.000 | -- | 2J1K | 155R | C | 0.052 | -- |
| 1DAN | 69S | U | 0.023 | -- | 2JEL | 62T | P | 0.000 | -- |
| 1DAN | 71K | U | 0.240 | -- | 2JEL | 70E | P | 2.727 | ** |
| 1DAN | 72K | U | 0.240 | -- | 2JEL | 83E | P | 0.000 | -- |
| 1DAN | 73T | U | 0.212 | -- | 2JEL | 85E | P | 0.000 | -- |
| 1DAN | 75K | U | 0.117 | -- | 2O3B | 24E | B | 5.472 | ** |
| 1DAN | 78T | U | 0.026 | -- | 2O3B | 74Q | B | 4.492 | ** |
| 1DAN | 79N | U | 0.025 | -- | 2O3B | 76W | B | 4.912 | ** |
| 1DAN | 80E | U | 0.025 | -- | 2PCC | 34D | A | 0.264 | -- |
| 1DAN | 82L | U | 0.080 | -- | 2PCC | 197V | A | 2.126 | ** |
| 1DAN | 85V | U | 0.110 | -- | 2PCC | 290E | A | 3.650 | ** |
| 1DAN | 87K | U | 0.017 | -- | 2QJA | 43T | C | 0.097 | -- |
| 1DAN | 89E | U | 0.094 | -- | 2QJA | 62P | C | 0.268 | -- |
| 1DAN | 90N | U | 0.094 | -- | 2SIC | 67M | I | 0.218 | -- |
| 1DAN | 91Y | U | 0.330 | -- | 2VLJ | 52D | E | 0.132 | -- |
| 1DAN | 101S | U | 0.318 | -- | 2VLJ | 95S | E | 0.035 | -- |
| 1DAN | 103T | U | 0.020 | -- | 2VLJ | 97Y | E | 0.232 | -- |
| 1DAN | 104V | U | 0.020 | -- | 2WPT | 30D | A | 0.132 | -- |
| 1DAN | 105N | U | 0.200 | -- | 2WPT | 34V | A | 3.459 | ** |
| 1DAN | 106R | U | 0.400 | -- | 2WPT | 38E | A | 4.501 | ** |
| 1DAN | 107K | U | 0.043 | -- | 2WPT | 39R | A | 0.240 | -- |
| 1DAN | 109T | U | 0.135 | -- | 2WPT | 47S | A | 2.424 | ** |
| 1DAN | 110D | U | 0.043 | -- | 2WPT | 53P | A | 2.926 | ** |
| 1DAN | 114E | U | 0.005 | -- | 2WPT | 67S | B | 0.134 | -- |
| 1DAN | 135L | H | 0.016 | -- | 2WPT | 71S | B | 0.095 | -- |
| 1DAN | 189K | H | 0.185 | -- | 2WPT | 77S | B | 0.067 | -- |
| 1DQJ | 20Y | C | 3.814 | ** | 2WPT | 80T | B | 0.376 | -- |
| 1DQJ | 21R | C | 2.271 | ** | 2WPT | 85Q | B | 0.384 | -- |
| 1DQJ | 96K | C | 5.978 | ** | 2WPT | 91V | B | 0.265 | -- |
| 1DQJ | 97K | C | 3.705 | ** | 3BK3 | 2L | C | 0.000 | -- |
| 1DQJ | 100S | C | 2.350 | ** | 3BN9 | 26I | B | 0.000 | -- |
| 1DQJ | 31N | A | 2.013 | ** | 3BN9 | 47D | B | 0.311 | -- |
| 1DQJ | 32N | A | 4.946 | ** | 3BN9 | 48R | B | 0.045 | -- |
| 1DQJ | 50Y | A | 2.678 | ** | 3BN9 | 50F | B | 0.045 | -- |
| 1DQJ | 91S | A | 2.725 | ** | 3BN9 | 51R | B | 0.072 | -- |
| 1DQJ | 33Y | B | 5.524 | ** | 3BN9 | 52Y | B | 0.019 | -- |
| 1DQJ | 50Y | B | 6.887 | ** | 3BN9 | 82R | B | 0.159 | -- |
| 1DQJ | 98W | B | 5.516 | ** | 3BN9 | 138H | B | 0.085 | -- |
| 1DVF | 32Y | A | 2.904 | ** | 3BN9 | 140Q | B | 0.133 | -- |
| 1DVF | 49Y | A | 2.003 | ** | 3BN9 | 144T | B | 0.291 | -- |
| 1DVF | 92W | A | 0.341 | -- | 3BN9 | 147L | B | 0.336 | -- |
| 1DVF | 52W | B | 4.143 | ** | 3BN9 | 163E | B | 0.373 | -- |
| 1DVF | 54D | B | 4.351 | ** | 3BN9 | 168Q | B | 0.035 | -- |
| 1DVF | 98E | B | 4.335 | ** | 3BN9 | 169Q | B | 2.509 | ** |
| 1DVF | 100D | B | 3.627 | ** | 3BN9 | 219R | B | 0.094 | -- |
| 1DVF | 33H | D | 2.790 | ** | 3BP8 | 115F | A | 0.033 | -- |
| 1DVF | 52D | D | 3.001 | ** | 3HFM | 20Y | Y | 5.400 | ** |
| 1DVF | 55N | D | 2.570 | ** | 3HFM | 21R | Y | 3.911 | ** |
| 1DVF | 101I | D | 2.681 | ** | 3HFM | 63W | Y | 0.319 | -- |
| 1DVF | 102Y | D | 4.686 | ** | 3HFM | 73R | Y | 0.331 | -- |
| 1DVF | 104Q | D | 2.185 | ** | 3HFM | 89T | Y | 0.000 | -- |
| 1DVF | 106R | D | 4.370 | ** | 3HFM | 93N | Y | 0.211 | -- |
| 1DVF | 49Y | C | 3.176 | ** | 3HFM | 96K | Y | 7.428 | ** |
| 1EAW | 45I | A | 0.194 | -- | 3HFM | 97K | Y | 5.705 | ** |
| 1EAW | 46D | A | 0.172 | -- | 3HFM | 98I | Y | 0.000 | -- |
| 1EAW | 51R | A | 0.231 | -- | 3HFM | 100S | Y | 0.268 | -- |
| 1EAW | 52Y | A | 0.079 | -- | 3HFM | 31N | L | 6.341 | ** |
| 1EAW | 82R | A | 0.150 | -- | 3HFM | 32N | L | 5.107 | ** |
| 1EAW | 90N | A | 0.308 | -- | 3HFM | 50Y | L | 6.493 | ** |
| 1EAW | 93T | A | 0.255 | -- | 3HFM | 96Y | L | 4.232 | ** |
| 1EAW | 138H | A | 0.014 | -- | 3HFM | 31S | H | 0.170 | -- |
| 1EAW | 140Q | A | 0.307 | -- | 3HFM | 32D | H | 2.538 | ** |
| 1EAW | 144T | A | 0.089 | -- | 3HFM | 33Y | H | 6.631 | ** |
| 1EAW | 169Q | A | 0.133 | -- | 3HFM | 50Y | H | 7.630 | ** |
| 1EAW | 214D | A | 2.228 | ** | 3HFM | 53Y | H | 3.250 | ** |
| 1EAW | 218Q | A | 0.144 | -- | 3HFM | 98W | H | 6.620 | ** |
| 1EAW | 219R | A | 0.089 | -- | 3NPS | 23Q | A | 0.026 | -- |
| 1EMV | 22N | A | 0.139 | -- | 3NPS | 45I | A | 0.332 | -- |
| 1EMV | 24D | A | 0.337 | -- | 3NPS | 46D | A | 0.340 | -- |
| 1EMV | 26S | A | 3.196 | ** | 3NPS | 50F | A | 0.220 | -- |
| 1EMV | 29E | A | 0.307 | -- | 3NPS | 51R | A | 0.138 | -- |
| 1EMV | 30E | A | 0.220 | -- | 3NPS | 82R | A | 0.152 | -- |
| 1EMV | 31L | A | 5.047 | ** | 3NPS | 90N | A | 0.253 | -- |
| 1EMV | 32V | A | 3.621 | ** | 3NPS | 140Q | A | 0.296 | -- |
| 1EMV | 33K | A | 0.192 | -- | 3NPS | 144T | A | 0.175 | -- |
| 1EMV | 35V | A | 2.865 | ** | 3NPS | 147L | A | 0.296 | -- |
| 1EMV | 39E | A | 2.083 | ** | 3NPS | 168Q | A | 0.059 | -- |
| 1EMV | 42T | A | 0.305 | -- | 3NPS | 218Q | A | 0.041 | -- |
| 1EMV | 43E | A | 0.213 | -- | 3NPS | 219R | A | 0.084 | -- |
| 1EMV | 46S | A | 0.007 | -- | 3NPS | 221K | A | 0.104 | -- |
| 1EMV | 48S | A | 2.187 | ** | 3SGB | 7K | I | 2.536 | ** |
| 1EMV | 49D | A | 5.915 | ** | 3SGB | 8P | I | 0.187 | -- |
| 1EMV | 52Y | A | 6.776 | ** | 3SGB | 11T | I | 3.363 | ** |
| 1EMV | 53Y | A | 6.447 | ** | 3SGB | 12L | I | 3.533 | ** |
| 1EMV | 67N | A | 0.278 | -- | 3SGB | 15R | I | 0.053 | -- |
| 1EMV | 74S | B | 0.241 | -- | 3SGB | 30N | I | 0.326 | -- |
| 1EMV | 75N | B | 2.334 | ** | 3SGB | 36V | I | 2.713 | ** |
| 1EMV | 77S | B | 0.233 | -- | 4CPA | 37V | I | 2.324 | ** |

** represents hot spots

-- represents non-hot spots

^&^ SKEMPI data is obtained by computing binding free energy change(∆∆G), so the value of the same residue of protein on other databases is a little different.

**5. Table** ^&^**S5 Test dataset derived from dbMPIKT**

| **PDB_ID** | **Residue** | **Chain** | **ΔΔGcom** | **Observed** | **PDB_ID** | **Residue** | **Chain** | **ΔΔGcom** | **Observed** |
| --- | --- | --- | --- | --- | --- | --- | --- | --- | --- |
| 1A4Y | W261 | A | 0.1 | -- | 3BDY | G97 | H | -0.1 | -- |
| 1A4Y | S289 | A | 0 | -- | 3BDY | D98 | H | 0 | -- |
| 1A4Y | K320 | A | -0.3 | -- | 3BDY | Y33 | H | 0.3 | -- |
| 1A4Y | E344 | A | 0.2 | -- | 3BDY | D27 | L | 0 | -- |
| 1AXI | F25 | A | -0.4 | -- | 3BDY | S52 | L | 0.1 | -- |
| 1BGS | E73 | A | 0.3 | -- | 3BDY | T93 | L | -0.3 | -- |
| 1BGS | Y29 | E | -0.1 | -- | 3BDY | T94 | L | 0 | -- |
| 1BGS | D39 | E | 7.7 | ** | 3BE1 | D31 | H | 0.39 | -- |
| 1BJ1 | F17 | V | 0 | -- | 3BE1 | T32 | H | -0.4 | -- |
| 1BJ1 | Y21 | V | 0 | -- | 3BE1 | Y33 | H | 2.4 | ** |
| 1BJ1 | Q79 | W | 0 | -- | 3BE1 | R50 | H | 2.4 | ** |
| 1BJ1 | M81 | W | 1 | ** | 3BE1 | Y52 | H | -0.8 | -- |
| 1BJ1 | R82 | W | 2.2 | ** | 3BE1 | T53 | H | 0.1 | -- |
| 1BJ1 | I83 | W | 3.69 | ** | 3BE1 | N54 | H | -0.8 | -- |
| 1BJ1 | H86 | W | 0.2 | -- | 3BE1 | R58 | H | 2.5 | ** |
| 1BJ1 | Q87 | W | 0 | -- | 3BE1 | G96 | H | 0.1 | -- |
| 1BJ1 | G88 | W | 2.76 | ** | 3BE1 | G97 | H | 0.3 | -- |
| 1BJ1 | H90 | W | 0 | -- | 3BE1 | D98 | H | -0.1 | -- |
| 1BJ1 | G92 | W | 3.69 | ** | 3BE1 | R27 | L | -0.2 | -- |
| 1CZ8 | F17 | V | 0 | -- | 3BE1 | S28 | L | 0.3 | -- |
| 1CZ8 | Y21 | V | 0 | -- | 3BE1 | S30 | L | -0.2 | -- |
| 1CZ8 | K48 | W | 0 | -- | 3BE1 | G31 | L | 0.3 | -- |
| 1CZ8 | M81 | W | 4.1 | ** | 3BE1 | Y32 | L | -0.8 | -- |
| 1CZ8 | H86 | W | 0 | -- | 3BE1 | G51 | L | -0.3 | -- |
| 1CZ8 | Q87 | W | 0 | -- | 3BE1 | S52 | L | 0.3 | -- |
| 1CZ8 | G88 | W | 2.67 | ** | 3BE1 | T93 | L | -0.4 | -- |
| 1CZ8 | H90 | W | 0 | -- | 3BIW | E577 | A | -1.24 | -- |
| 1CZ8 | G92 | W | 4.1 | ** | 3BN9 | D96 | A | 6.7 | ** |
| 1DFJ | W259 | I | 2.2 | ** | 3BN9 | F97 | A | 6.7 | ** |
| 1DFJ | Y430 | I | 5.9 | ** | 3BN9 | T28 | H | 0.3 | -- |
| 1DFJ | D431 | I | 3.6 | ** | 3BN9 | S30 | H | 0.36 | -- |
| 1DFJ | Y433 | I | 2.6 | ** | 3BN9 | T98 | H | 0.3 | -- |
| 1DFJ | I455 | I | 0.3 | -- | 3BN9 | Y99 | H | 0 | -- |
| 1DVF | I101 | D | 2.71 | ** | 3BN9 | Q100 | H | -0.06 | -- |
| 1DVF | Y102 | D | 4.79 | ** | 3K2M | Y36 | D | 3.7 | ** |
| 1F47 | E17 | B | 0.14 | -- | 3K2M | R38 | D | 2.5 | ** |
| 1F47 | S20 | B | -0.43 | -- | 3K2M | E52 | D | 2.5 | ** |
| 1F47 | L22 | B | 0.37 | -- | 3K2M | W80 | D | 4 | ** |
| 1F47 | L25 | B | 0.3 | -- | 3K2M | M88 | D | 4 | ** |
| 1F47 | P27 | B | -1.26 | -- | 3K2M | Y87 | D | 4 | ** |
| 1F47 | E28 | B | -0.36 | -- | 3MBW | G111 | A | 0.14 | -- |
| 1ILP | M1 | C | 0.38 | -- | 3NGB | I30 | B | -0.01 | -- |
| 1ILP | W2 | C | 2.71 | ** | 3NGB | R53 | B | -0.27 | -- |
| 1ILP | D3 | C | 2 | ** | 3NGB | G54 | B | -1.28 | -- |
| 1ILP | F4 | C | 2.06 | ** | 3NGB | P62 | B | -0.05 | -- |
| 1ILP | D5 | C | 2.74 | ** | 3NGB | Q64 | B | -0.35 | -- |
| 1ILP | D6 | C | 2.72 | ** | 3NGB | Y74 | B | -0.21 | -- |
| 1ILP | G7 | C | -0.41 | -- | 3NGB | D99 | B | 0.05 | -- |
| 1ILP | M8 | C | 0.45 | ** | 3NGB | Y100 | B | 1.42 | -- |
| 1ILP | P9 | C | 2.97 | -- | 3NGB | V3 | C | -0.78 | -- |
| 1ILP | P10 | C | 1.49 | -- | 3NGB | Q27 | C | -0.24 | -- |
| 1ILP | D12 | C | 2.2 | -- | 3NGB | S30 | C | -0.15 | -- |
| 1ILP | E13 | C | 1.56 | -- | 3NGB | Y91 | C | 2.01 | ** |
| 1ILP | D14 | C | 2.59 | ** | 3NPS | Q23 | A | 0.03 | -- |
| 1ILP | S16 | C | -0.26 | -- | 3NPS | I45 | A | -0.34 | -- |
| 1ILP | P17 | C | 2.24 | ** | 3NPS | D46 | A | 0.34 | -- |
| 1ILP | A11 | C | 0.12 | -- | 3NPS | R48 | A | -1.07 | -- |
| 1JTD | Y105 | A | 2.29 | ** | 3NPS | F50 | A | 0.22 | -- |
| 1JTD | E110 | A | 0.04 | -- | 3NPS | R51 | A | 0.14 | -- |
| 1JTD | K111 | A | 2.18 | ** | 3NPS | R82 | A | -0.15 | -- |
| 1JTD | M129 | A | 2.56 | ** | 3NPS | N90 | A | 0.26 | -- |
| 1JTD | V216 | A | 0.04 | -- | 3NPS | Q140 | A | 0.3 | -- |
| 1JTD | M272 | A | 0.28 | -- | 3NPS | T144 | A | 0.18 | -- |
| 1JTD | T57 | B | 0.286329001 | -- | 3NPS | L147 | A | 0.3 | -- |
| 1JTD | Y73 | B | 3.079220598 | ** | 3NPS | Q168 | A | -0.06 | -- |
| 1JTD | F74 | B | 2.492900776 | ** | 3NPS | Q218 | A | -0.04 | -- |
| 1JTD | W152 | B | 3.180346571 | ** | 3NPS | R219 | A | -0.08 | -- |
| 1JTD | S169 | B | 0.147386307 | -- | 3NPS | K221 | A | -0.1 | -- |
| 1JTD | D170 | B | -0.537627453 | -- | 3Q3J | Y114 | B | 0.3 | -- |
| 1JTD | Y191 | B | 3.221927375 | ** | 3SE8 | R30 | H | -0.04 | -- |
| 1JTD | D206 | B | -1.039385927 | -- | 3SE8 | L54 | H | 0.05 | -- |
| 1JTD | F209 | B | 2.15025303 | ** | 3SE8 | W55 | H | -0.01 | -- |
| 1JTD | I229 | B | 2.823561223 | ** | 3SE8 | G56 | H | 0.35 | -- |
| 1JTD | F230 | B | 3.870446379 | ** | 3SE8 | R72 | H | 2.65 | ** |
| 1JTD | W269 | B | 5.67769409 | ** | 3SE8 | Q76 | H | 0.09 | -- |
| 1JTG | E36 | B | 3.15 | ** | 3SE8 | P78 | H | 0.01 | -- |
| 1K8R | D38 | A | 3.1 | ** | 3SE8 | P81 | H | -0.08 | -- |
| 1K8R | R41 | A | 3.1 | ** | 3SE8 | D110 | H | -0.38 | -- |
| 1KTZ | F30 | B | 3 | ** | 3SE8 | D114 | H | -0.24 | -- |
| 1KTZ | N47 | B | 0.3 | -- | 3SE8 | E1 | L | -0.57 | -- |
| 1KTZ | S49 | B | 0.3 | -- | 3SE8 | Q27 | L | -0.22 | -- |
| 1KTZ | S52 | B | 0.2 | -- | 3SE8 | N30 | L | 0.2 | -- |
| 1KTZ | H79 | B | 0.3 | -- | 3SE8 | F90 | L | -0.87 | -- |
| 1KTZ | E119 | B | 0.3 | -- | 3SE9 | V3 | L | -0.51 | -- |
| 1KTZ | D32 | B | 0.1 | -- | 3SE9 | F97 | L | 0.02 | -- |
| 1MHP | R31 | H | 0.2 | -- | 3W2D | Y46 | A | 0.1 | -- |
| 1MHP | T33 | H | 0.1 | -- | 3W2D | K71 | A | 0.22 | -- |
| 1MHP | S35 | H | 0.32 | -- | 4BFI | G113 | A | 0.3 | -- |
| 1MHP | T50 | H | -0.26 | -- | 4BJ5 | E47 | A | 0.1 | -- |
| 1MHP | S52 | H | -0.34 | -- | 4BKL | S31 | A | 0.206169551 | -- |
| 1MHP | G53 | H | 0.21 | -- | 4BPX | E44 | A | -0.848852262 | -- |
| 1MHP | G54 | H | -0.12 | -- | 4BPX | Y54 | A | 0.119803162 | -- |
| 1MHP | H56 | H | 0.1 | -- | 4BPX | R56 | A | -0.04620941 | -- |
| 1MHP | Y58 | H | -0.5 | -- | 4BPX | K77 | A | 0.161406463 | -- |
| 1MHP | Y59 | H | 0.15 | -- | 4BPX | D109 | A | -0.065751418 | -- |
| 1MHP | L60 | H | -0.12 | -- | 4BPX | D111 | A | -1.382782255 | -- |
| 1MHP | K64 | H | -0.07 | -- | 4BPX | D114 | A | -0.262674184 | -- |
| 1MHP | F99 | H | 0 | -- | 4BPX | D116 | A | -0.075096306 | -- |
| 1MHP | G100 | H | 0.1 | -- | 4BPX | D306 | A | -0.198013192 | -- |
| 1MHP | G102 | H | 0.8 | -- | 4BPX | H315 | A | -0.138967672 | -- |
| 1MHP | A25 | L | 0 | -- | 4BPX | K318 | A | -0.065751418 | -- |
| 1MHP | V29 | L | 0.1 | -- | 4BPX | H324 | A | -0.06508953 | -- |
| 1MHP | H31 | L | 0.3 | -- | 4BPX | R183 | A | 0.259762714 | -- |
| 1MHP | M32 | L | 0.2 | -- | 4BPX | F178 | A | -0.871107185 | -- |
| 1MHP | T50 | L | -0.1 | -- | 4BPX | Y223 | A | -0.142726217 | -- |
| 1MHP | L53 | L | -0.4 | -- | 4BPX | R227 | A | -0.38532035 | -- |
| 1MHP | S91 | L | 0.3 | -- | 4BPX | F104 | B | 2.221011929 | ** |
| 1MHP | G92 | L | 0.2 | -- | 4BPX | R107 | B | 2.046757271 | ** |
| 1MHP | N93 | L | 0.1 | -- | 4BPX | L108 | B | 2.226577421 | ** |
| 1MHP | P94 | L | -0.1 | -- | 4FZA | E138 | B | -1.053772163 | -- |
| 1MLC | N32 | A | 0 | -- | 4HMY | Q71 | C | 0.1 | -- |
| 1MLC | N92 | A | -1.25 | -- | 4HSA | W67 | A | 0.155508697 | -- |
| 1MLC | T31 | B | 0.13 | -- | 4HSA | H86 | A | 0.255926322 | -- |
| 1MLC | S57 | B | -0.38 | -- | 4HSA | Y85 | A | 0.396253833 | -- |
| 1MLC | T58 | B | -0.56 | -- | 4IOF | F530 | A | 2.63574435 | ** |
| 1MLC | K65 | B | 0.02 | -- | 4J2L | W37 | C | 2.278266248 | ** |
| 1MLC | T28 | B | -0.15 | -- | 4J2L | L40 | C | 0.34 | -- |
| 1MTN | T311 | D | 0.2 | -- | 4JEU | R171 | A | -0.261751662 | -- |
| 1MTN | P313 | D | -0.1 | -- | 4JEU | R39 | A | 0.162304397 | -- |
| 1MTN | I319 | D | 0.1 | -- | 4KRM | R30 | B | 2.18117592 | ** |
| 1MTN | R320 | D | 0.3 | -- | 4L72 | N229 | A | -6.883113358 | -- |
| 1MTN | V334 | D | 0 | -- | 4LRX | H56 | A | 0.3204415 | -- |
| 1MTN | G336 | D | 0.9 | -- | 4MYW | I80 | A | -1.519990714 | -- |
| 1MTN | R339 | D | 0.2 | -- | 4NM8 | I18 | B | 0.109878921 | -- |
| 1N8Z | D28 | A | -0.28 | -- | 4NM8 | D19 | B | 0.162095779 | -- |
| 1N8Z | N30 | A | 0.06 | -- | 4NM8 | R25 | B | 0.3 | -- |
| 1N8Z | S50 | A | -0.07 | -- | 4NM8 | T32 | B | -0.050251223 | -- |
| 1N8Z | S52 | A | -0.31 | -- | 4NM8 | Q34 | B | 0.222295079 | -- |
| 1N8Z | F53 | A | -0.43 | -- | 4NM8 | L38 | B | -0.615714011 | -- |
| 1N8Z | R66 | A | 0.22 | -- | 4NZW | K96 | A | 0.076847885 | -- |
| 1N8Z | H91 | A | 0.1 | -- | 4NZW | E59 | A | 0.331030211 | -- |
| 1N8Z | Y92 | A | 0.36 | -- | 4NZW | E134 | B | 0.363211628 | -- |
| 1N8Z | T94 | A | -0.12 | -- | 4O27 | E58 | B | 0.377875746 | -- |
| 1N8Z | D31 | B | 0.25 | -- | 4O27 | E138 | B | 0.306891969 | -- |
| 1N8Z | T32 | B | 0.38 | -- | 4OFY | M56 | A | 0.071916142 | -- |
| 1N8Z | Y33 | B | -0.09 | -- | 4OFY | F60 | A | 4.594586354 | ** |
| 1N8Z | Y52 | B | 0.23 | -- | 4OFY | S107 | A | 0.129886137 | -- |
| 1N8Z | N54 | B | -0.15 | -- | 4OFY | E108 | A | 0.270374364 | -- |
| 1N8Z | W95 | B | 8 | ** | 4OFY | Q53 | D | 2.859845996 | ** |
| 1N8Z | D98 | B | -0.69 | -- | 4OFY | L61 | D | 2.557084998 | ** |
| 1N8Z | F100 | B | -0.05 | -- | 4OFY | Q105 | D | 4.635478149 | ** |
| 1N8Z | Y102 | B | 0.22 | -- | 4OFY | R115 | D | 3.410814856 | ** |
| 1N8Z | R50 | B | 8 | ** | 4OZG | N36 | E | -0.05005153 | -- |
| 1NVU | G12 | R | -4.24 | -- | 4OZG | Y40 | E | 0.3 | -- |
| 1S78 | L525 | A | -0.009560217 | -- | 4OZG | Y55 | F | -0.59377684 | -- |
| 1S78 | S551 | A | -1.319173166 | -- | 4OZG | N63 | F | -0.083171578 | -- |
| 1S78 | V552 | A | 0.047260556 | -- | 4OZG | L103 | F | -0.2 | -- |
| 1S78 | F555 | A | 0.045616392 | -- | 4P23 | V72 | C | 0 | -- |
| 1U0S | V103 | Y | 0.01 | -- | 4P23 | K75 | C | 0.2 | -- |
| 1U7F | D493 | B | 2.7 | ** | 4P23 | E59 | D | 0.1 | -- |
| 1VFB | N31 | A | 0.17 | -- | 4P23 | Y60 | D | 0.2 | -- |
| 1VFB | Y32 | A | 0.16 | -- | 4P23 | S63 | D | 0 | -- |
| 1VFB | L46 | A | 0.13 | -- | 4P23 | Q64 | D | 0.1 | -- |
| 1VFB | Y50 | A | 0.23 | -- | 4P23 | P65 | D | 0.1 | -- |
| 1VFB | T52 | A | 0.18 | -- | 4P23 | E66 | D | 0.1 | -- |
| 1VFB | T53 | A | 0.13 | -- | 4P23 | I67 | D | 0.2 | -- |
| 1VFB | W28 | C | 0.33 | -- | 4P23 | E69 | D | 0.2 | -- |
| 1VFB | A31 | C | 0 | -- | 4P23 | R70 | D | 0.2 | -- |
| 1VFB | A32 | C | 0 | -- | 4P23 | R72 | D | 0 | -- |
| 1VFB | V99 | C | -1.2 | -- | 4P23 | D76 | D | 0 | -- |
| 1VFB | D101 | C | 8 | ** | 4P23 | T77 | D | 0 | -- |
| 1VFB | I58 | C | 0.12 | -- | 4P23 | R80 | D | 0 | -- |
| 1YCR | L26 | B | 2.39 | ** | 4P23 | H81 | D | 0.2 | -- |
| 1YCS | P177 | A | -1.29 | -- | 4P23 | E84 | D | 0.1 | -- |
| 1YCS | R248 | A | -24.31 | -- | 4P4Q | E32 | A | 0.16 | -- |
| 1YCS | R273 | A | -3.44 | -- | 4P4Q | D34 | A | -0.9 | -- |
| 1YCS | D281 | A | -0.903 | -- | 4P4Q | E35 | A | 0.17 | -- |
| 1YY9 | S26 | C | -0.2 | -- | 4P4Q | A193 | A | 0 | -- |
| 1YY9 | T31 | C | -0.52 | -- | 4P4Q | V197 | A | 2.1 | ** |
| 1YY9 | N93 | C | -0.74 | -- | 4P4Q | E290 | A | -0.8 | -- |
| 1YY9 | N56 | D | -0.06 | -- | 4P4Q | E291 | A | -0.1 | -- |
| 1YY9 | T61 | D | -0.06 | -- | 4P4Q | A81 | B | 0 | -- |
| 2KSO | M13 | A | -0.9 | -- | 4P5T | Q57 | C | 0 | -- |
| 2NYY | S902 | A | -0.23 | -- | 4P5T | Q61 | C | 0 | -- |
| 2NYY | K903 | A | 0.29 | -- | 4P5T | V72 | C | 0 | -- |
| 2NYY | L919 | A | 2.59 | -- | 4P5T | K75 | C | 0.3 | -- |
| 2NYY | E920 | A | 2.84 | -- | 4P5T | E59 | D | 0.1 | -- |
| 2NYY | K923 | A | -0.14 | -- | 4P5T | Y60 | D | 0.1 | -- |
| 2NYY | F953 | A | 4.06 | ** | 4P5T | S63 | D | 0 | -- |
| 2NYY | N954 | A | 0.1 | -- | 4P5T | Q64 | D | 0 | -- |
| 2NYY | I956 | A | -0.01 | -- | 4P5T | P65 | D | 0 | -- |
| 2NYY | K1056 | A | -0.03 | -- | 4P5T | E66 | D | 0.2 | -- |
| 2NYY | D1058 | A | 0.01 | -- | 4P5T | I67 | D | 0 | -- |
| 2NYY | D1062 | A | 2.53 | ** | 4P5T | E69 | D | 0.1 | -- |
| 2NYY | H1064 | A | 7.32 | ** | 4P5T | R70 | D | 0.1 | -- |
| 2NYY | R1294 | A | 0.3 | -- | 4P5T | R72 | D | 0 | -- |
| 2NYY | S955 | A | 0 | -- | 4P5T | D76 | D | 0 | -- |
| 2NYY | S28 | C | 0.32 | -- | 4P5T | T77 | D | 0.2 | -- |
| 2NYY | D30 | C | 0.25 | -- | 4P5T | R80 | D | 0 | -- |
| 2NYY | S31 | C | -0.36 | -- | 4P5T | H81 | D | 0.2 | -- |
| 2NYY | H34 | C | 0.1 | -- | 4P5T | E84 | D | 0 | -- |
| 2NYY | K30 | D | 0.07 | -- | 4PWX | K69 | A | 0.202390236 | -- |
| 2NYY | Y31 | D | 0.07 | -- | 4PWX | K72 | A | 0.259762714 | -- |
| 2NYY | M34 | D | -0.06 | -- | 4PWX | R75 | A | 0.359161384 | -- |
| 2NYY | Y57 | D | 0.13 | -- | 4PWX | E253 | A | 0.2 | -- |
| 2NZ9 | Q915 | A | 0.1 | -- | 4PWX | E304 | A | 0.18 | -- |
| 2NZ9 | F917 | A | -0.05 | -- | 4PWX | E31 | C | -0.686585163 | -- |
| 2NZ9 | L919 | A | 2.28 | ** | 4PWX | H33 | C | -0.35435029 | -- |
| 2NZ9 | N918 | A | 2.16 | ** | 4PWX | Q34 | C | -0.178471183 | -- |
| 2NZ9 | E920 | A | 2.77 | ** | 4PWX | R45 | C | 0.335073125 | -- |
| 2NZ9 | K923 | A | -0.3 | -- | 4PWX | E81 | C | 0.103958986 | -- |
| 2NZ9 | F953 | A | 3.34 | ** | 4QTI | R58 | U | 3 | ** |
| 2NZ9 | N954 | A | -0.15 | -- | 4QTI | R89 | U | 3 | ** |
| 2NZ9 | S955 | A | -0.08 | -- | 4QTI | R91 | U | 3 | ** |
| 2NZ9 | I956 | A | 0.07 | -- | 4QTI | Y92 | U | 3 | ** |
| 2NZ9 | K1056 | A | -0.01 | -- | 4QTI | E94 | U | 3 | ** |
| 2NZ9 | D1058 | A | -0.03 | -- | 4QTI | Q114 | U | 3 | ** |
| 2NZ9 | R1061 | A | 0.29 | -- | 4RA0 | G32 | C | 0.1 | -- |
| 2NZ9 | D1062 | A | 2.34 | ** | 4RA0 | D87 | C | -0.5 | -- |
| 2NZ9 | T1063 | A | 2.37 | ** | 4RA0 | V92 | C | -0.6 | -- |
| 2NZ9 | H1064 | A | 7.42 | ** | 4RA0 | G127 | C | -0.01 | -- |
| 2NZ9 | R1294 | A | 0.39 | -- | 4U6H | N27 | E | -1.204738919 | -- |
| 2NZ9 | S902 | A | -0.12 | -- | 4UWQ | R416 | A | -1 | -- |
| 2PTC | K15 | I | 10 | ** | 4X4M | K173 | E | -0.75 | -- |
| 2X0B | Y4 | B | 2.87 | ** | 4X4M | H174 | E | -0.63 | -- |
| 2X0B | I5 | B | 3.69 | ** | 4X4M | R175 | E | -1.92 | -- |
| 2X0B | H6 | B | 4.97 | ** | 4Y61 | Y273 | A | -0.044929235 | -- |
| 2X0B | F8 | B | 6.02 | ** | 4Y61 | R114 | B | -0.154916273 | -- |
| 2X0B | H9 | B | 6.28 | ** | 4YEB | A313 | A | 0.065582469 | -- |
| 2X0B | L10 | B | 6.44 | ** | 4YEB | R117 | B | 0.274118663 | -- |
| 2X0B | V11 | B | 3.63 | ** | 4YEB | F160 | B | -1.687660563 | -- |
| 2X0B | I12 | B | 7.36 | ** | 4YFD | R75 | A | 0.296819816 | -- |
| 2X0B | H13 | B | 6.52 | ** | 4YFD | R196 | A | 0.259762714 | -- |
| 2X0B | E15 | B | 3.68 | ** | 4YFD | D30 | B | -0.024196036 | -- |
| 2X0B | K55 | B | 3.47 | ** | 5C6T | Y280 | A | 3.211234335 | ** |
| 2X0B | L56 | B | 3.47 | ** | 5C6T | N281 | A | 0.284111691 | -- |
| 2X0B | R57 | B | 2.7 | ** | 5C6T | T283 | A | 0.33809636 | -- |
| 2X0B | F67 | B | 3.13 | ** | 5C6T | N284 | A | 0.3614206 | -- |
| 2X0B | R71 | B | 5.59 | ** | 5C6T | N286 | A | -0.367425539 | -- |
| 2X0B | Y73 | B | 2.44 | ** | 5C6T | F297 | A | 2.760639263 | ** |
| 2X0B | V119 | B | -1.7 | -- | 5C6T | R31 | H | 0.311017395 | -- |
| 2X0B | K122 | B | -1.7 | -- | 5C6T | S32 | H | -0.868535725 | -- |
| 2X0B | D123 | B | 4.43 | ** | 5C6T | Y109 | H | 0.3614206 | -- |
| 2X0B | Q318 | B | 2.83 | ** | 5C6T | R51 | L | 0.122701557 | -- |
| 2X0B | K326 | B | 5.11 | ** | 5CYK | E481 | A | -0.348404816 | -- |
| 2X0B | L327 | B | 2.51 | ** | 5CYK | T484 | A | -0.646101524 | -- |
| 3BDY | D31 | H | 0.2 | -- | 5CYK | R486 | A | -0.152317007 | -- |
| 3BDY | K30 | H | 0.2 | -- | 5CYK | V488 | A | -0.152317007 | -- |
| 3BDY | T32 | H | -0.4 | -- | 5E6P | Y1806 | A | 0.364633185 | -- |
| 3BDY | R50 | H | -0.3 | -- | 5E6P | A1832 | A | 0.118505006 | -- |
| 3BDY | N54 | H | -0.2 | -- | 5E6P | A1833 | A | 0.60511707 | -- |
| 3BDY | T53 | H | -0.5 | -- | 5E6P | I66 | B | 0.278580821 | -- |
| 3BDY | Y56 | H | 0.2 | -- | 5E6P | R88 | B | -0.6 | -- |
| 3BDY | R58 | H | -0.2 | -- | 5K39 | D39 | B | 4 | ** |
| 3BDY | G96 | H | 0.2 | -- | 5K39 | L83 | B | -3 | -- |

** represents hot spots

-- represents non-hot spots

^& This data set comes from our database dbMPIKT^

**6. Table S6 Test dataset derived from Mix data-set**

Similarly, we removed the same proteins. That is removed the same data from three data-sets(BID, SKEMPI and dbMPIKT) to make up a new mixed data-set to validate our model.

| **PDB_ID** | **Residue** | **Chain** | **ΔΔG_com_** | **Observed** | **PDB_ID** | **Residue** | **Chain** | **ΔΔG_com_** | **Observed** |
| --- | --- | --- | --- | --- | --- | --- | --- | --- | --- |
| 1A22 | 3T | A | 0.05 | -- | 1NMB | 103Y | H | 2.14 | ** |
| 1A22 | 7S | A | 0.34 | -- | 1N8Z | D28 | A | -0.28 | -- |
| 1A22 | 8R | A | 0.2 | -- | 1N8Z | N30 | A | 0.06 | -- |
| 1A22 | 9L | A | 0.04 | -- | 1N8Z | S50 | A | -0.07 | -- |
| 1A22 | 12N | A | 0.1 | -- | 1N8Z | S52 | A | -0.31 | -- |
| 1A22 | 14M | A | 0 | -- | 1N8Z | F53 | A | -0.43 | -- |
| 1A22 | 15L | A | 0.15 | -- | 1N8Z | R66 | A | 0.22 | -- |
| 1A22 | 16R | A | 0.238 | -- | 1N8Z | H91 | A | 0.1 | -- |
| 1A22 | 19R | A | 0.051 | -- | 1N8Z | Y92 | A | 0.36 | -- |
| 1A22 | 21H | A | 0.155 | -- | 1N8Z | T94 | A | -0.12 | -- |
| 1A22 | 22Q | A | 0.22 | -- | 1N8Z | D31 | B | 0.25 | -- |
| 1A22 | 25F | A | 0.229 | -- | 1N8Z | T32 | B | 0.38 | -- |
| 1A22 | 26D | A | 0.211 | -- | 1N8Z | Y33 | B | -0.09 | -- |
| 1A22 | 42Y | A | 0.221 | -- | 1N8Z | Y52 | B | 0.23 | -- |
| 1A22 | 46Q | A | 0.191 | -- | 1N8Z | N54 | B | -0.15 | -- |
| 1A22 | 51S | A | 0.348 | -- | 1N8Z | W95 | B | 8 | ** |
| 1A22 | 55S | A | 0.11 | -- | 1N8Z | D98 | B | -0.69 | -- |
| 1A22 | 57S | A | 0.2 | -- | 1N8Z | F100 | B | -0.05 | -- |
| 1A22 | 59P | A | 0.38 | -- | 1N8Z | Y102 | B | 0.22 | -- |
| 1A22 | 62S | A | 0.155 | -- | 1N8Z | R50 | B | 8 | ** |
| 1A22 | 63N | A | 0.314 | -- | 1NVU | G12 | R | -4.24 | -- |
| 1A22 | 69Q | A | 0.05 | -- | 1S78 | L525 | A | -0.009560217 | -- |
| 1A22 | 73L | A | 0.2 | -- | 1S78 | S551 | A | -1.319173166 | -- |
| 1A22 | 153Y | A | 0.348 | -- | 1S78 | V552 | A | 0.047260556 | -- |
| 1A22 | 156R | A | 0.278 | -- | 1S78 | F555 | A | 0.045616392 | -- |
| 1A22 | 157K | A | 0.155 | -- | 1UB4 | F453 | C | N | -- |
| 1A22 | 161K | A | 2.014 | ** | 1UB4 | L455 | C | S | ** |
| 1A22 | 167R | A | 2.424 | ** | 1UB4 | L458 | C | S | ** |
| 1A22 | 169V | A | 0 | -- | 1U0S | V103 | Y | 0.01 | -- |
| 1A22 | 170Q | A | 0.27 | -- | 1U7F | D493 | B | 2.7 | ** |
| 1A22 | 173S | A | 0.05 | -- | 1PPF | 14P | I | 0.122 | -- |
| 1A22 | 175E | A | 0.012 | -- | 1PPF | 17T | I | 3.145 | ** |
| 1A22 | 176G | A | 0.34 | -- | 1PPF | 18L | I | 3.541 | ** |
| 1A22 | 177S | A | 0.2 | -- | 1PPF | 20Y | I | 3.166 | ** |
| 1A22 | 180F | A | 0.191 | -- | 1PPF | 21R | I | 0.205 | -- |
| 1A22 | 2K | B | 0.24 | -- | 1PPF | 42V | I | 2.581 | ** |
| 1A22 | 5K | B | 0.24 | -- | 1R0R | 13L | I | 3.775 | ** |
| 1A22 | 7R | B | 0.269 | -- | 1R0R | 14E | I | 2.06 | ** |
| 1A22 | 9P | B | 0.26 | -- | 1R0R | 15Y | I | 5.398 | ** |
| 1A22 | 15S | B | 0.015 | -- | 1R0R | 16R | I | 0.095 | -- |
| 1A22 | 19T | B | 0.07 | -- | 1R0R | 27G | I | 3.081 | ** |
| 1A22 | 31N | B | 0.011 | -- | 1R0R | 31N | I | 0.033 | -- |
| 1A22 | 32T | B | 0.077 | -- | 1REW | 38F | A | 2.123 | ** |
| 1A22 | 33Q | B | 0.194 | -- | 1REW | 39P | A | 2.088 | ** |
| 1A22 | 34E | B | 0.172 | -- | 1REW | 42D | A | 0.065 | -- |
| 1A22 | 36T | B | 0.025 | -- | 1REW | 77S | A | 0.193 | -- |
| 1A22 | 38E | B | 0.079 | -- | 1REW | 89L | A | 0.176 | -- |
| 1A22 | 39W | B | 0.05 | -- | 1REW | 38F | B | 2.123 | ** |
| 1A22 | 40K | B | 0.144 | -- | 1REW | 39P | B | 2.088 | ** |
| 1A22 | 50E | B | 0.178 | -- | 1REW | 42D | B | 0.065 | -- |
| 1A22 | 54Y | B | 0.199 | -- | 1REW | 77S | B | 0.193 | -- |
| 1A22 | 56N | B | 0.255 | -- | 1REW | 89L | B | 0.176 | -- |
| 1A22 | 57S | B | 0.161 | -- | 1REW | 55Q | C | 2.657 | ** |
| 1A22 | 59F | B | 0.015 | -- | 1S1Q | 42F | A | 0.196 | -- |
| 1A22 | 61S | B | 0.028 | -- | 1S1Q | 73W | A | 0.276 | -- |
| 1A22 | 65P | B | 2.965 | ** | 1TM1 | 39T | I | 2.896 | ** |
| 1A22 | 67C | B | 0 | -- | 1TM1 | 41E | I | 2.53 | ** |
| 1A22 | 69K | B | 0.043 | -- | 1TM1 | 42Y | I | 2.579 | ** |
| 1A22 | 78D | B | 0.21 | -- | 1TM1 | 46R | I | 3.96 | ** |
| 1A22 | 79E | B | 0.057 | -- | 1TM1 | 48R | I | 3.689 | ** |
| 1A22 | 80K | B | 0.13 | -- | 1TM1 | 51V | I | 0.025 | -- |
| 1A22 | 81C | B | 0 | -- | 1VFB | 92W | A | 3.357 | ** |
| 1A22 | 83S | B | 0.237 | -- | 1VFB | 93S | A | 0.343 | -- |
| 1A22 | 88V | B | 0.046 | -- | 1VFB | 19N | C | 0.396 | -- |
| 1A22 | 89Q | B | 0.03 | -- | 1VFB | 24S | C | 2.11 | ** |
| 1A22 | 107D | B | 0.314 | -- | 1VFB | 121Q | C | 2.66 | ** |
| 1A22 | 111R | B | 0.314 | -- | 1VFB | 124I | C | 2.662 | ** |
| 1A22 | 113E | B | 0.314 | -- | 1VFB | 125R | C | 2.615 | ** |
| 1A22 | 116R | B | 0.062 | -- | 1XD3 | 8L | B | 2.738 | ** |
| 1A22 | 120I | B | 2.164 | ** | 1XD3 | 24E | B | 0.246 | -- |
| 1A22 | 121Q | B | 0.009 | -- | 1XD3 | 27K | B | 0.064 | -- |
| 1A22 | 122K | B | 0.027 | -- | 1XD3 | 44I | B | 0.272 | -- |
| 1A22 | 128E | B | 0.083 | -- | 1XD3 | 52D | B | 0.246 | -- |
| 1A22 | 130E | B | 0.079 | -- | 1XD3 | 58D | B | 0.246 | -- |
| 1A22 | 134K | B | 0 | -- | 1Z7X | 263W | W | 2.211 | ** |
| 1A22 | 135E | B | 0.199 | -- | 1Z7X | 434Y | W | 6.786 | ** |
| 1A22 | 138E | B | 0.12 | -- | 1Z7X | 435D | W | 5.414 | ** |
| 1A22 | 140K | B | 0.12 | -- | 1Z7X | 437Y | W | 4.93 | ** |
| 1A22 | 142K | B | 0.12 | -- | 1Z7X | 459I | W | 0.337 | -- |
| 1A22 | 145D | B | 0.12 | -- | 1YCS | P177 | A | -1.29 | -- |
| 1A22 | 149T | B | 0.202 | -- | 1YCS | R248 | A | -24.31 | -- |
| 1A22 | 150T | B | 0.097 | -- | 1YCS | R273 | A | -3.44 | -- |
| 1A22 | 166R | B | 0.056 | -- | 1YCS | D281 | A | -0.903 | -- |
| 1A22 | 168R | B | 0.19 | -- | 1YY9 | S26 | C | -0.2 | -- |
| 1A22 | 172R | B | 0.268 | -- | 1YY9 | T31 | C | -0.52 | -- |
| 1A22 | 173N | B | 0.298 | -- | 1YY9 | N93 | C | -0.74 | -- |
| 1A22 | 174S | B | 0.034 | -- | 1YY9 | N56 | D | -0.06 | -- |
| 1A22 | 179E | B | 0.108 | -- | 1YY9 | T61 | D | -0.06 | -- |
| 1AK4 | 85P | D | 2.449 | ** | 2HHB | Y35 | D | N | -- |
| 1AK4 | 86V | D | 2.355 | ** | 2NMB | M99 | A | S | ** |
| 1AK4 | 87H | D | 2.373 | ** | 2NMB | C150 | A | S | ** |
| 1AK4 | 89G | D | 3.44 | ** | 2NMB | Y2 | B | I | -- |
| 1AK4 | 90P | D | 3.535 | ** | 2NMB | I3 | B | I | -- |
| 1AK4 | 93P | D | 2.046 | ** | 2FTL | 12G | I | 4.346 | ** |
| 1AXI | F25 | A | -0.4 | -- | 2FTL | 15K | I | 10.289 | ** |
| 1BGS | E73 | A | 0.3 | -- | 2FTL | 18I | I | 4.969 | ** |
| 1BGS | Y29 | E | -0.1 | -- | 2FTL | 36G | I | 2.191 | ** |
| 1BGS | D39 | E | 7.7 | ** | 2G2U | 36F | B | 2.762 | ** |
| 1BJ1 | F17 | V | 0 | -- | 2G2U | 50Y | B | 2.2 | ** |
| 1BJ1 | Y21 | V | 0 | -- | 2G2U | 53Y | B | 2.301 | ** |
| 1BJ1 | Q79 | W | 0 | -- | 2G2U | 73E | B | 2.088 | ** |
| 1BJ1 | M81 | W | 1 | ** | 2G2U | 74K | B | 0.217 | -- |
| 1BJ1 | R82 | W | 2.2 | ** | 2G2U | 112W | B | 0.274 | -- |
| 1BJ1 | I83 | W | 3.69 | ** | 2G2U | 142F | B | 0.276 | -- |
| 1BJ1 | H86 | W | 0.2 | -- | 2G2U | 144R | B | 0.342 | -- |
| 1BJ1 | Q87 | W | 0 | -- | 2I9B | 129R | E | 0.287 | -- |
| 1BJ1 | G88 | W | 2.76 | ** | 2I9B | 134R | E | 0.363 | -- |
| 1BJ1 | H90 | W | 0 | -- | 2J0T | 2T | D | 4.387 | ** |
| 1BJ1 | G92 | W | 3.69 | ** | 2J0T | 68S | D | 3.694 | ** |
| 1CDL | F12 | A | N | -- | 2J1K | 155R | C | 0.052 | -- |
| 1CDL | F19 | A | W | -- | 2JEL | 62T | P | 0 | -- |
| 1CDL | F92 | A | S | ** | 2JEL | 70E | P | 2.727 | ** |
| 1CDL | K799 | E | N | -- | 2JEL | 83E | P | 0 | -- |
| 1CDL | W800 | E | S | ** | 2JEL | 85E | P | 0 | -- |
| 1CDL | K802 | E | I | -- | 2O3B | 24E | B | 5.472 | ** |
| 1CDL | R808 | E | I | -- | 2O3B | 74Q | B | 4.492 | ** |
| 1CDL | I810 | E | S | ** | 2O3B | 76W | B | 4.912 | ** |
| 1CDL | L813 | E | S | ** | 2PCC | 34D | A | 0.264 | -- |
| 1CDL | G804 | E | S | ** | 2PCC | 197V | A | 2.126 | ** |
| 1CDL | G811 | E | I | -- | 2PCC | 290E | A | 3.65 | ** |
| 1CDL | R812 | E | S | ** | 2QJA | 43T | C | 0.097 | -- |
| 1CBW | 11T | I | 0.22 | -- | 2QJA | 62P | C | 0.268 | -- |
| 1CBW | 13P | I | 0.056 | -- | 2SIC | 67M | I | 0.218 | -- |
| 1CBW | 15K | I | 2.091 | ** | 2VLJ | 52D | E | 0.132 | -- |
| 1CBW | 19I | I | 0.141 | -- | 2VLJ | 95S | E | 0.035 | -- |
| 1CBW | 20R | I | 0.35 | -- | 2VLJ | 97Y | E | 0.232 | -- |
| 1CBW | 33F | I | 0.141 | -- | 2WPT | 30D | A | 0.132 | -- |
| 1CBW | 34V | I | 0.051 | -- | 2WPT | 34V | A | 3.459 | ** |
| 1CBW | 39R | I | 0.22 | -- | 2WPT | 38E | A | 4.501 | ** |
| 1CBW | 46K | I | 0.141 | -- | 2WPT | 39R | A | 0.24 | -- |
| 1CHO | 10K | I | 0.179 | -- | 2WPT | 47S | A | 2.424 | ** |
| 1CHO | 11P | I | 0.375 | -- | 2WPT | 53P | A | 2.926 | ** |
| 1CHO | 14T | I | 4.179 | ** | 2WPT | 67S | B | 0.134 | -- |
| 1CHO | 15L | I | 6.271 | ** | 2WPT | 71S | B | 0.095 | -- |
| 1CHO | 16E | I | 2.301 | ** | 2WPT | 77S | B | 0.067 | -- |
| 1CHO | 17Y | I | 2.508 | ** | 2WPT | 80T | B | 0.376 | -- |
| 1CHO | 18R | I | 3.147 | ** | 2WPT | 85Q | B | 0.384 | -- |
| 1CHO | 39V | I | 5.095 | ** | 2WPT | 91V | B | 0.265 | -- |
| 1CZ8 | F17 | V | 0 | -- | 2KSO | M13 | A | -0.9 | -- |
| 1CZ8 | Y21 | V | 0 | -- | 2NYY | S902 | A | -0.23 | -- |
| 1CZ8 | K48 | W | 0 | -- | 2NYY | K903 | A | 0.29 | -- |
| 1CZ8 | M81 | W | 4.1 | ** | 2NYY | L919 | A | 2.59 | -- |
| 1CZ8 | H86 | W | 0 | -- | 2NYY | E920 | A | 2.84 | -- |
| 1CZ8 | Q87 | W | 0 | -- | 2NYY | K923 | A | -0.14 | -- |
| 1CZ8 | G88 | W | 2.67 | ** | 2NYY | F953 | A | 4.06 | ** |
| 1CZ8 | H90 | W | 0 | -- | 2NYY | N954 | A | 0.1 | -- |
| 1CZ8 | G92 | W | 4.1 | ** | 2NYY | I956 | A | -0.01 | -- |
| 1DDM | I144 | A | S | ** | 2NYY | K1056 | A | -0.03 | -- |
| 1DDM | E145 | A | S | ** | 2NYY | D1058 | A | 0.01 | -- |
| 1DDM | K146 | A | I | -- | 2NYY | D1062 | A | 2.53 | ** |
| 1DDM | S148 | A | N | -- | 2NYY | H1064 | A | 7.32 | ** |
| 1DDM | C150 | A | S | ** | 2NYY | R1294 | A | 0.3 | -- |
| 1DDM | R165 | A | I | -- | 2NYY | S955 | A | 0 | -- |
| 1DDM | C198 | A | S | ** | 2NYY | S28 | C | 0.32 | -- |
| 1DDM | F2 | B | I | -- | 2NYY | D30 | C | 0.25 | -- |
| 1DDM | S3 | B | S | ** | 2NYY | S31 | C | -0.36 | -- |
| 1DDM | N4 | B | S | ** | 2NYY | H34 | C | 0.1 | -- |
| 1DDM | M5 | B | S | ** | 2NYY | K30 | D | 0.07 | -- |
| 1DDM | S6 | B | S | ** | 2NYY | Y31 | D | 0.07 | -- |
| 1DDM | F7 | B | S | ** | 2NYY | M34 | D | -0.06 | -- |
| 1DDM | E8 | B | W | -- | 2NYY | Y57 | D | 0.13 | -- |
| 1DDM | F10 | B | W | -- | 2NZ9 | Q915 | A | 0.1 | -- |
| 1DFJ | K7 | E | S | ** | 2NZ9 | F917 | A | -0.05 | -- |
| 1DFJ | W259 | I | 2.2 | ** | 2NZ9 | L919 | A | 2.28 | ** |
| 1DFJ | Y430 | I | 5.9 | ** | 2NZ9 | N918 | A | 2.16 | ** |
| 1DFJ | D431 | I | 3.6 | ** | 2NZ9 | E920 | A | 2.77 | ** |
| 1DFJ | Y433 | I | 2.6 | ** | 2NZ9 | K923 | A | -0.3 | -- |
| 1DFJ | I455 | I | 0.3 | -- | 2NZ9 | F953 | A | 3.34 | ** |
| 1DVA | L32 | H | S | ** | 2NZ9 | N954 | A | -0.15 | -- |
| 1DVA | L34 | H | S | ** | 2NZ9 | S955 | A | -0.08 | -- |
| 1DVA | N37 | H | N | -- | 2NZ9 | I956 | A | 0.07 | -- |
| 1DVA | I65 | H | N | -- | 2NZ9 | K1056 | A | -0.01 | -- |
| 1DVA | V67 | H | N | -- | 2NZ9 | D1058 | A | -0.03 | -- |
| 1DVA | E70 | H | W | -- | 2NZ9 | R1061 | A | 0.29 | -- |
| 1DVA | L73 | H | N | -- | 2NZ9 | D1062 | A | 2.34 | ** |
| 1DVA | S74 | H | N | -- | 2NZ9 | T1063 | A | 2.37 | ** |
| 1DVA | E75 | H | N | -- | 2NZ9 | H1064 | A | 7.42 | ** |
| 1DVA | H76 | H | S | ** | 2NZ9 | R1294 | A | 0.39 | -- |
| 1DVA | E80 | H | N | -- | 2NZ9 | S902 | A | -0.12 | -- |
| 1DVA | S82 | H | N | -- | 2PTC | K15 | I | 10 | ** |
| 1DVA | L144 | H | N | -- | 2X0B | Y4 | B | 2.87 | ** |
| 1DVA | L153 | H | W | -- | 2X0B | I5 | B | 3.69 | ** |
| 1DVA | G38 | H | I | -- | 2X0B | H6 | B | 4.97 | ** |
| 1DVA | A1 | X | N | -- | 2X0B | F8 | B | 6.02 | ** |
| 1DVA | D5 | X | W | -- | 2X0B | H9 | B | 6.28 | ** |
| 1DVA | Q14 | X | N | -- | 2X0B | L10 | B | 6.44 | ** |
| 1DVA | V16 | X | N | -- | 2X0B | V11 | B | 3.63 | ** |
| 1DVA | L2 | X | S | ** | 2X0B | I12 | B | 7.36 | ** |
| 1DVA | R7 | X | W | -- | 2X0B | H13 | B | 6.52 | ** |
| 1DVA | V8 | X | I | -- | 2X0B | E15 | B | 3.68 | ** |
| 1DVA | D9 | X | I | -- | 2X0B | K55 | B | 3.47 | ** |
| 1DVA | W11 | X | S | ** | 2X0B | L56 | B | 3.47 | ** |
| 1DVA | Y12 | X | S | ** | 2X0B | R57 | B | 2.7 | ** |
| 1DVA | F15 | X | S | ** | 2X0B | F67 | B | 3.13 | ** |
| 1DZI | N154 | A | I | -- | 2X0B | R71 | B | 5.59 | ** |
| 1DZI | Y157 | A | S | ** | 2X0B | Y73 | B | 2.44 | ** |
| 1DZI | Q215 | A | S | ** | 2X0B | V119 | B | -1.7 | -- |
| 1DZI | D219 | A | I | -- | 2X0B | K122 | B | -1.7 | -- |
| 1DZI | L220 | A | N | -- | 2X0B | D123 | B | 4.43 | ** |
| 1DZI | T221 | A | S | ** | 2X0B | Q318 | B | 2.83 | ** |
| 1DZI | E256 | A | N | -- | 2X0B | K326 | B | 5.11 | ** |
| 1DZI | H258 | A | I | -- | 2X0B | L327 | B | 2.51 | ** |
| 1DAN | 135L | H | 0.016 | -- | 3SAK | E8 | A | W | -- |
| 1DAN | 189K | H | 0.185 | -- | 3SAK | F10 | A | S | ** |
| 1DQJ | 20Y | C | 3.814 | ** | 3SAK | T11 | A | I | -- |
| 1DQJ | 21R | C | 2.271 | ** | 3SAK | L12 | A | S | ** |
| 1DQJ | 96K | C | 5.978 | ** | 3SAK | Q13 | A | W | -- |
| 1DQJ | 97K | C | 3.705 | ** | 3SAK | I14 | A | S | ** |
| 1DQJ | 100S | C | 2.35 | ** | 3SAK | R15 | A | I | -- |
| 1DQJ | 31N | A | 2.013 | ** | 3SAK | R17 | A | W | -- |
| 1DQJ | 32N | A | 4.946 | ** | 3SAK | R19 | A | S | ** |
| 1DQJ | 50Y | A | 2.678 | ** | 3SAK | F20 | A | S | ** |
| 1DQJ | 91S | A | 2.725 | ** | 3SAK | F23 | A | S | ** |
| 1DQJ | 33Y | B | 5.524 | ** | 3SAK | L26 | A | S | ** |
| 1DQJ | 50Y | B | 6.887 | ** | 3SAK | N27 | A | I | -- |
| 1DQJ | 98W | B | 5.516 | ** | 3SAK | L30 | A | S | ** |
| 1DVF | 49Y | C | 3.176 | ** | 3SAK | D34 | A | W | -- |
| 1DX5 | I24 | N | N | -- | 3BK3 | 2L | C | 0 | -- |
| 1DX5 | F34 | N | I | -- | 3BN9 | 26I | B | 0 | -- |
| 1DX5 | K36 | N | W | -- | 3BN9 | 47D | B | 0.311 | -- |
| 1DX5 | P37 | N | W | -- | 3BN9 | 48R | B | 0.045 | -- |
| 1DX5 | Q38 | N | W | -- | 3BN9 | 50F | B | 0.045 | -- |
| 1DX5 | E39 | N | N | -- | 3BN9 | 51R | B | 0.072 | -- |
| 1DX5 | L65 | N | W | -- | 3BN9 | 52Y | B | 0.019 | -- |
| 1DX5 | R67 | N | S | ** | 3BN9 | 82R | B | 0.159 | -- |
| 1DX5 | E80 | N | S | ** | 3BN9 | 138H | B | 0.085 | -- |
| 1DX5 | K81 | N | W | -- | 3BN9 | 140Q | B | 0.133 | -- |
| 1DX5 | I82 | N | I | -- | 3BN9 | 144T | B | 0.291 | -- |
| 1DX5 | M84 | N | N | -- | 3BN9 | 147L | B | 0.336 | -- |
| 1DX5 | K110 | N | N | -- | 3BN9 | 163E | B | 0.373 | -- |
| 1DX5 | K235 | N | N | -- | 3BN9 | 168Q | B | 0.035 | -- |
| 1EBP | F93 | A | S | ** | 3BN9 | 169Q | B | 2.509 | ** |
| 1EBP | M150 | A | S | ** | 3BN9 | 219R | B | 0.094 | -- |
| 1EBP | T151 | A | W | -- | 3BN9 | D96 | A | 6.7 | ** |
| 1EBP | F205 | A | S |  | 3BN9 | F97 | A | 6.7 | ** |
| 1EBP | L11 | C | N | -- | 3BN9 | T28 | H | 0.3 | -- |
| 1EBP | T12 | C | I | -- | 3BN9 | S30 | H | 0.36 | -- |
| 1EBP | W13 | C | S | ** | 3BN9 | T98 | H | 0.3 | -- |
| 1EBP | G9 | C | I | -- | 3BN9 | Y99 | H | 0 | -- |
| 1EBP | P10 | C | I | -- | 3BN9 | Q100 | H | -0.06 | -- |
| 1ES7 | V26 | A | I | -- | 3BP8 | 115F | A | 0.033 | -- |
| 1ES7 | W31 | A | S | ** | 3NPS | 23Q | A | 0.026 | -- |
| 1ES7 | P50 | A | I | -- | 3NPS | 45I | A | 0.332 | -- |
| 1ES7 | F49 | A | I | -- | 3NPS | 46D | A | 0.34 | -- |
| 1EAW | 45I | A | 0.194 | -- | 3NPS | 50F | A | 0.22 | -- |
| 1EAW | 46D | A | 0.172 | -- | 3NPS | 51R | A | 0.138 | -- |
| 1EAW | 51R | A | 0.231 | -- | 3NPS | 82R | A | 0.152 | -- |
| 1EAW | 52Y | A | 0.079 | -- | 3NPS | 90N | A | 0.253 | -- |
| 1EAW | 82R | A | 0.15 | -- | 3NPS | 140Q | A | 0.296 | -- |
| 1EAW | 90N | A | 0.308 | -- | 3NPS | 144T | A | 0.175 | -- |
| 1EAW | 93T | A | 0.255 | -- | 3NPS | 147L | A | 0.296 | -- |
| 1EAW | 138H | A | 0.014 | -- | 3NPS | 168Q | A | 0.059 | -- |
| 1EAW | 140Q | A | 0.307 | -- | 3NPS | 218Q | A | 0.041 | -- |
| 1EAW | 144T | A | 0.089 | -- | 3NPS | 219R | A | 0.084 | -- |
| 1EAW | 169Q | A | 0.133 | -- | 3NPS | 221K | A | 0.104 | -- |
| 1EAW | 214D | A | 2.228 | ** | 3SGB | 7K | I | 2.536 | ** |
| 1EAW | 218Q | A | 0.144 | -- | 3SGB | 8P | I | 0.187 | -- |
| 1EAW | 219R | A | 0.089 | -- | 3SGB | 11T | I | 3.363 | ** |
| 1EMV | 22N | A | 0.139 | -- | 3SGB | 12L | I | 3.533 | ** |
| 1EMV | 24D | A | 0.337 | -- | 3SGB | 15R | I | 0.053 | -- |
| 1EMV | 26S | A | 3.196 | ** | 3SGB | 30N | I | 0.326 | -- |
| 1EMV | 29E | A | 0.307 | -- | 3SGB | 36V | I | 2.713 | ** |
| 1EMV | 30E | A | 0.22 | -- | 3BDY | D31 | H | 0.2 | -- |
| 1EMV | 31L | A | 5.047 | ** | 3BDY | K30 | H | 0.2 | -- |
| 1EMV | 32V | A | 3.621 | ** | 3BDY | T32 | H | -0.4 | -- |
| 1EMV | 33K | A | 0.192 | -- | 3BDY | R50 | H | -0.3 | -- |
| 1EMV | 35V | A | 2.865 | ** | 3BDY | N54 | H | -0.2 | -- |
| 1EMV | 39E | A | 2.083 | ** | 3BDY | T53 | H | -0.5 | -- |
| 1EMV | 42T | A | 0.305 | -- | 3BDY | Y56 | H | 0.2 | -- |
| 1EMV | 43E | A | 0.213 | -- | 3BDY | R58 | H | -0.2 | -- |
| 1EMV | 46S | A | 0.007 | -- | 3BDY | G96 | H | 0.2 | -- |
| 1EMV | 48S | A | 2.187 | ** | 3BDY | G97 | H | -0.1 | -- |
| 1EMV | 49D | A | 5.915 | ** | 3BDY | D98 | H | 0 | -- |
| 1EMV | 52Y | A | 6.776 | ** | 3BDY | Y33 | H | 0.3 | -- |
| 1EMV | 53Y | A | 6.447 | ** | 3BDY | D27 | L | 0 | -- |
| 1EMV | 67N | A | 0.278 | -- | 3BDY | S52 | L | 0.1 | -- |
| 1EMV | 74S | B | 0.241 | -- | 3BDY | T93 | L | -0.3 | -- |
| 1EMV | 75N | B | 2.334 | ** | 3BDY | T94 | L | 0 | -- |
| 1EMV | 77S | B | 0.233 | -- | 3BE1 | D31 | H | 0.39 | -- |
| 1EMV | 84S | B | 0.109 | -- | 3BE1 | T32 | H | -0.4 | -- |
| 1EMV | 86F | B | 6.044 | ** | 3BE1 | Y33 | H | 2.4 | ** |
| 1EMV | 87T | B | 0.158 | -- | 3BE1 | R50 | H | 2.4 | ** |
| 1EMV | 92Q | B | 0.278 | -- | 3BE1 | Y52 | H | -0.8 | -- |
| 1FCC | E27 | C | S | ** | 3BE1 | T53 | H | 0.1 | -- |
| 1FCC | K28 | C | W | -- | 3BE1 | N54 | H | -0.8 | -- |
| 1FCC | K31 | C | S | ** | 3BE1 | R58 | H | 2.5 | ** |
| 1FCC | N35 | C | I | -- | 3BE1 | G96 | H | 0.1 | -- |
| 1FCC | D40 | C | N | -- | 3BE1 | G97 | H | 0.3 | -- |
| 1FCC | E42 | C | N | -- | 3BE1 | D98 | H | -0.1 | -- |
| 1FCC | W43 | C | S | ** | 3BE1 | R27 | L | -0.2 | -- |
| 1FE8 | R963 | A | N | -- | 3BE1 | S28 | L | 0.3 | -- |
| 1FE8 | E987 | A | N | -- | 3BE1 | S30 | L | -0.2 | -- |
| 1FE8 | H990 | A | N | -- | 3BE1 | G31 | L | 0.3 | -- |
| 1FE8 | H1023 | A | N | -- | 3BE1 | Y32 | L | -0.8 | -- |
| 1FOE | G54 | B | S | ** | 3BE1 | G51 | L | -0.3 | -- |
| 1FOE | S41 | B | I | -- | 3BE1 | S52 | L | 0.3 | -- |
| 1F47 | 8I | A | 2.515 | ** | 3BE1 | T93 | L | -0.4 | -- |
| 1F47 | 9P | A | 0.058 | -- | 3BIW | E577 | A | -1.24 | -- |
| 1F47 | 11F | A | 2.444 | ** | 3K2M | Y36 | D | 3.7 | ** |
| 1F47 | 12L | A | 2.294 | ** | 3K2M | R38 | D | 2.5 | ** |
| 1F47 | 14K | A | 0.043 | -- | 3K2M | E52 | D | 2.5 | ** |
| 1F47 | 15Q | A | 0.046 | -- | 3K2M | W80 | D | 4 | ** |
| 1F47 | E17 | B | 0.14 | -- | 3K2M | M88 | D | 4 | ** |
| 1F47 | S20 | B | -0.43 | -- | 3K2M | Y87 | D | 4 | ** |
| 1F47 | L22 | B | 0.37 | -- | 3MBW | G111 | A | 0.14 | -- |
| 1F47 | L25 | B | 0.3 | -- | 3NGB | I30 | B | -0.01 | -- |
| 1F47 | P27 | B | -1.26 | -- | 3NGB | R53 | B | -0.27 | -- |
| 1F47 | E28 | B | -0.36 | -- | 3NGB | G54 | B | -1.28 | -- |
| 1FC2 | 26F | C | 0.014 | -- | 3NGB | P62 | B | -0.05 | -- |
| 1FC2 | 27I | C | 3.729 | ** | 3NGB | Q64 | B | -0.35 | -- |
| 1FFW | 23H | B | 0.034 | -- | 3NGB | Y74 | B | -0.21 | -- |
| 1FFW | 44D | B | 0.074 | -- | 3NGB | D99 | B | 0.05 | -- |
| 1FFW | 49D | B | 0.096 | -- | 3NGB | Y100 | B | 1.42 | -- |
| 1FFW | 55C | B | 0.204 | -- | 3NGB | V3 | C | -0.78 | -- |
| 1FFW | 56F | B | 3.644 | ** | 3NGB | Q27 | C | -0.24 | -- |
| 1FAK | Q37 | T | W | -- | 3NGB | S30 | C | -0.15 | -- |
| 1FAK | K41 | T | N | -- | 3NGB | Y91 | C | 2.01 | ** |
| 1FAK | S42 | T | N | -- | 3Q3J | Y114 | B | 0.3 | -- |
| 1FAK | D44 | T | W | -- | 3SE8 | R30 | H | -0.04 | -- |
| 1FAK | Y94 | T | W | -- | 3SE8 | L54 | H | 0.05 | -- |
| 1FAK | K15 | T | N | -- | 3SE8 | W55 | H | -0.01 | -- |
| 1FAK | T17 | T | N | -- | 3SE8 | G56 | H | 0.35 | -- |
| 1FAK | N18 | T | N | -- | 3SE8 | R72 | H | 2.65 | ** |
| 1FAK | K20 | T | S | ** | 3SE8 | Q76 | H | 0.09 | -- |
| 1FAK | I22 | T | W | -- | 3SE8 | P78 | H | 0.01 | -- |
| 1FAK | E24 | T | W | -- | 3SE8 | P81 | H | -0.08 | -- |
| 1FAK | S47 | T | N | -- | 3SE8 | D110 | H | -0.38 | -- |
| 1FAK | S48 | T | N | -- | 3SE8 | D114 | H | -0.24 | -- |
| 1FAK | F50 | T | N | -- | 3SE8 | E1 | L | -0.57 | -- |
| 1FAK | D58 | T | S | ** | 3SE8 | Q27 | L | -0.22 | -- |
| 1FAK | E128 | T | N | -- | 3SE8 | N30 | L | 0.2 | -- |
| 1FAK | L133 | T | N | -- | 3SE8 | F90 | L | -0.87 | -- |
| 1FAK | R135 | T | N | -- | 3SE9 | V3 | L | -0.51 | -- |
| 1FAK | F140 | T | I | -- | 3SE9 | F97 | L | 0.02 | -- |
| 1FAK | T203 | T | N | -- | 3W2D | Y46 | A | 0.1 | -- |
| 1FAK | V207 | T | N | -- | 3W2D | K71 | A | 0.22 | -- |
| 1GL4 | D427 | A | S | ** | 4BFI | G113 | A | 0.3 | -- |
| 1GL4 | H429 | A | S | ** | 4BJ5 | E47 | A | 0.1 | -- |
| 1GL4 | Y431 | A | S | ** | 4BKL | S31 | A | 0.206169551 | -- |
| 1GL4 | Y440 | A | I | -- | 4BPX | E44 | A | -0.848852262 | -- |
| 1GL4 | E616 | A | S | ** | 4BPX | Y54 | A | 0.119803162 | -- |
| 1GL4 | R620 | A | S | ** | 4BPX | R56 | A | -0.04620941 | -- |
| 1GL4 | R403 | A | I | -- | 4BPX | K77 | A | 0.161406463 | -- |
| 1G3I | D438 | A | S | ** | 4BPX | D109 | A | -0.065751418 | -- |
| 1G3I | L439 | A | S | ** | 4BPX | D111 | A | -1.382782255 | -- |
| 1G3I | R441 | A | S | ** | 4BPX | D114 | A | -0.262674184 | -- |
| 1G3I | F442 | A | S | ** | 4BPX | D116 | A | -0.075096306 | -- |
| 1G3I | I443 | A | S | ** | 4BPX | D306 | A | -0.198013192 | -- |
| 1G3I | L444 | A | S | ** | 4BPX | H315 | A | -0.138967672 | -- |
| 1GCQ | 18P | C | 0.121 | -- | 4BPX | K318 | A | -0.065751418 | -- |
| 1GCQ | 19P | C | 0.085 | -- | 4BPX | H324 | A | -0.06508953 | -- |
| 1IHB | N101 | B | N | -- | 4BPX | R183 | A | 0.259762714 | -- |
| 1IHB | R133 | B | W | -- | 4BPX | F178 | A | -0.871107185 | -- |
| 1IHB | H135 | B | W | -- | 4BPX | Y223 | A | -0.142726217 | -- |
| 1IHB | K136 | B | I | -- | 4BPX | R227 | A | -0.38532035 | -- |
| 1H9D | 94N | B | 2.11 | ** | 4BPX | F104 | B | 2.221011929 | ** |
| 1IAR | 6T | A | 0.104 | -- | 4BPX | R107 | B | 2.046757271 | ** |
| 1IAR | 8Q | A | 0.022 | -- | 4BPX | L108 | B | 2.226577421 | ** |
| 1IAR | 11I | A | 0.069 | -- | 4CPA | 37V | I | 2.324 | ** |
| 1IAR | 15N | A | 0.034 | -- | 4FZA | E138 | B | -1.053772163 | -- |
| 1IAR | 16S | A | 0.183 | -- | 4HMY | Q71 | C | 0.1 | -- |
| 1IAR | 19E | A | 0.32 | -- | 4HSA | W67 | A | 0.155508697 | -- |
| 1IAR | 77K | A | 0.154 | -- | 4HSA | H86 | A | 0.255926322 | -- |
| 1IAR | 78Q | A | 0.125 | -- | 4HSA | Y85 | A | 0.396253833 | -- |
| 1IAR | 82F | A | 0.086 | -- | 4IOF | F530 | A | 2.63574435 | ** |
| 1IAR | 84K | A | 0.345 | -- | 4J2L | W37 | C | 2.278266248 | ** |
| 1IAR | 88R | A | 3.753 | ** | 4J2L | L40 | C | 0.34 | -- |
| 1ILP | M1 | C | 0.38 | -- | 4JEU | R171 | A | -0.261751662 | -- |
| 1ILP | W2 | C | 2.71 | ** | 4JEU | R39 | A | 0.162304397 | -- |
| 1ILP | D3 | C | 2 | ** | 4KRM | R30 | B | 2.18117592 | ** |
| 1ILP | F4 | C | 2.06 | ** | 4L72 | N229 | A | -6.883113358 | -- |
| 1ILP | D5 | C | 2.74 | ** | 4LRX | H56 | A | 0.3204415 | -- |
| 1ILP | D6 | C | 2.72 | ** | 4MYW | I80 | A | -1.519990714 | -- |
| 1ILP | G7 | C | -0.41 | -- | 4NM8 | I18 | B | 0.109878921 | -- |
| 1ILP | M8 | C | 0.45 | ** | 4NM8 | D19 | B | 0.162095779 | -- |
| 1ILP | P9 | C | 2.97 | -- | 4NM8 | R25 | B | 0.3 | -- |
| 1ILP | P10 | C | 1.49 | -- | 4NM8 | T32 | B | -0.050251223 | -- |
| 1ILP | D12 | C | 2.2 | -- | 4NM8 | Q34 | B | 0.222295079 | -- |
| 1ILP | E13 | C | 1.56 | -- | 4NM8 | L38 | B | -0.615714011 | -- |
| 1ILP | D14 | C | 2.59 | ** | 4NZW | K96 | A | 0.076847885 | -- |
| 1ILP | S16 | C | -0.26 | -- | 4NZW | E59 | A | 0.331030211 | -- |
| 1ILP | P17 | C | 2.24 | ** | 4NZW | E134 | B | 0.363211628 | -- |
| 1ILP | A11 | C | 0.12 | -- | 4O27 | E58 | B | 0.377875746 | -- |
| 1JAT | E55 | A | S | ** | 4O27 | E138 | B | 0.306891969 | -- |
| 1JAT | F8 | B | S | ** | 4OFY | M56 | A | 0.071916142 | -- |
| 1JCK | 90Y | B | 2.595 | ** | 4OFY | F60 | A | 4.594586354 | ** |
| 1JCK | 91V | B | 2.232 | ** | 4OFY | S107 | A | 0.129886137 | -- |
| 1JCK | 176F | B | 2.133 | ** | 4OFY | E108 | A | 0.270374364 | -- |
| 1JRH | 37K | I | 3.714 | ** | 4OFY | Q53 | D | 2.859845996 | ** |
| 1JRH | 38N | I | 0.17 | -- | 4OFY | L61 | D | 2.557084998 | ** |
| 1JRH | 39Y | I | 3.527 | ** | 4OFY | Q105 | D | 4.635478149 | ** |
| 1JRH | 40G | I | 4.45 | ** | 4OFY | R115 | D | 3.410814856 | ** |
| 1JRH | 42K | I | 3.386 | ** | 4OZG | N36 | E | -0.05005153 | -- |
| 1JRH | 43N | I | 4.299 | ** | 4OZG | Y40 | E | 0.3 | -- |
| 1JRH | 44S | I | 0.378 | -- | 4OZG | Y55 | F | -0.59377684 | -- |
| 1JRH | 69N | I | 0.2 | -- | 4OZG | N63 | F | -0.083171578 | -- |
| 1JRH | 71L | I | 2.959 | ** | 4OZG | L103 | F | -0.2 | -- |
| 1JRH | 72W | I | 4.429 | ** | 4P23 | V72 | C | 0 | -- |
| 1JRH | 73V | I | 0.214 | -- | 4P23 | K75 | C | 0.2 | -- |
| 1JRH | 74R | I | 0.393 | -- | 4P23 | E59 | D | 0.1 | -- |
| 1JRH | 88K | I | 0.312 | -- | 4P23 | Y60 | D | 0.2 | -- |
| 1JRH | 92W | L | 2.818 | ** | 4P23 | S63 | D | 0 | -- |
| 1JRH | 94T | L | 0.385 | -- | 4P23 | Q64 | D | 0.1 | -- |
| 1JRH | 54W | H | 2.686 | ** | 4P23 | P65 | D | 0.1 | -- |
| 1JRH | 55W | H | 2.421 | ** | 4P23 | E66 | D | 0.1 | -- |
| 1JRH | 103F | H | 0 | -- | 4P23 | I67 | D | 0.2 | -- |
| 1JTD | Y105 | A | 2.29 | ** | 4P23 | E69 | D | 0.2 | -- |
| 1JTD | E110 | A | 0.04 | -- | 4P23 | R70 | D | 0.2 | -- |
| 1JTD | K111 | A | 2.18 | ** | 4P23 | R72 | D | 0 | -- |
| 1JTD | M129 | A | 2.56 | ** | 4P23 | D76 | D | 0 | -- |
| 1JTD | V216 | A | 0.04 | -- | 4P23 | T77 | D | 0 | -- |
| 1JTD | M272 | A | 0.28 | -- | 4P23 | R80 | D | 0 | -- |
| 1JTD | T57 | B | 0.286329001 | -- | 4P23 | H81 | D | 0.2 | -- |
| 1JTD | Y73 | B | 3.079220598 | ** | 4P23 | E84 | D | 0.1 | -- |
| 1JTD | F74 | B | 2.492900776 | ** | 4P4Q | E32 | A | 0.16 | -- |
| 1JTD | W152 | B | 3.180346571 | ** | 4P4Q | D34 | A | -0.9 | -- |
| 1JTD | S169 | B | 0.147386307 | -- | 4P4Q | E35 | A | 0.17 | -- |
| 1JTD | D170 | B | -0.537627453 | -- | 4P4Q | A193 | A | 0 | -- |
| 1JTD | Y191 | B | 3.221927375 | ** | 4P4Q | V197 | A | 2.1 | ** |
| 1JTD | D206 | B | -1.039385927 | -- | 4P4Q | E290 | A | -0.8 | -- |
| 1JTD | F209 | B | 2.15025303 | ** | 4P4Q | E291 | A | -0.1 | -- |
| 1JTD | I229 | B | 2.823561223 | ** | 4P4Q | A81 | B | 0 | -- |
| 1JTD | F230 | B | 3.870446379 | ** | 4P5T | Q57 | C | 0 | -- |
| 1JTD | W269 | B | 5.67769409 | ** | 4P5T | Q61 | C | 0 | -- |
| 1JTG | E36 | B | 3.15 | ** | 4P5T | V72 | C | 0 | -- |
| 1JPP | K345 | B | S | ** | 4P5T | K75 | C | 0.3 | -- |
| 1JPP | K354 | B | N | -- | 4P5T | E59 | D | 0.1 | -- |
| 1JPP | W383 | B | S | ** | 4P5T | Y60 | D | 0.1 | -- |
| 1JPP | R386 | B | I | -- | 4P5T | S63 | D | 0 | -- |
| 1JPP | K435 | B | I | -- | 4P5T | Q64 | D | 0 | -- |
| 1JPP | R469 | B | I | -- | 4P5T | P65 | D | 0 | -- |
| 1JPP | H470 | B | I | -- | 4P5T | E66 | D | 0.2 | -- |
| 1NUN | D76 | A | I | -- | 4P5T | I67 | D | 0 | -- |
| 1NUN | R78 | A | I | -- | 4P5T | E69 | D | 0.1 | -- |
| 1NUN | R155 | A | I | -- | 4P5T | R70 | D | 0.1 | -- |
| 1K4U | R368 | P | S | ** | 4P5T | R72 | D | 0 | -- |
| 1K4U | L373 | P | W | -- | 4P5T | D76 | D | 0 | -- |
| 1K4U | I374 | P | S | ** | 4P5T | T77 | D | 0.2 | -- |
| 1K4U | R377 | P | W | -- | 4P5T | R80 | D | 0 | -- |
| 1K4U | T382 | P | I | -- | 4P5T | H81 | D | 0.2 | -- |
| 1KTZ | 64R | A | 2.883 | ** | 4P5T | E84 | D | 0 | -- |
| 1KTZ | 3L | B | 2.27 | ** | 4PWX | K69 | A | 0.202390236 | -- |
| 1KTZ | 6F | B | 3.425 | ** | 4PWX | K72 | A | 0.259762714 | -- |
| 1KTZ | 26I | B | 2.342 | ** | 4PWX | R75 | A | 0.359161384 | -- |
| 1K8R | D38 | A | 3.1 | ** | 4PWX | E253 | A | 0.2 | -- |
| 1K8R | R41 | A | 3.1 | ** | 4PWX | E304 | A | 0.18 | -- |
| 1LQB | M561 | D | N | -- | 4PWX | E31 | C | -0.686585163 | -- |
| 1LQB | L562 | D | N | -- | 4PWX | H33 | C | -0.35435029 | -- |
| 1LFD | 35K | A | 0.258 | -- | 4PWX | Q34 | C | -0.178471183 | -- |
| 1LFD | 43D | A | 0.28 | -- | 4PWX | R45 | C | 0.335073125 | -- |
| 1LFD | 44E | A | 0.246 | -- | 4PWX | E81 | C | 0.103958986 | -- |
| 1MQ8 | T206 | B | S | ** | 4QTI | R58 | U | 3 | ** |
| 1MAH | 327Y | A | 0.386 | -- | 4QTI | R89 | U | 3 | ** |
| 1MHP | R31 | H | 0.2 | -- | 4QTI | R91 | U | 3 | ** |
| 1MHP | T33 | H | 0.1 | -- | 4QTI | Y92 | U | 3 | ** |
| 1MHP | S35 | H | 0.32 | -- | 4QTI | E94 | U | 3 | ** |
| 1MHP | T50 | H | -0.26 | -- | 4QTI | Q114 | U | 3 | ** |
| 1MHP | S52 | H | -0.34 | -- | 4RA0 | G32 | C | 0.1 | -- |
| 1MHP | G53 | H | 0.21 | -- | 4RA0 | D87 | C | -0.5 | -- |
| 1MHP | G54 | H | -0.12 | -- | 4RA0 | V92 | C | -0.6 | -- |
| 1MHP | H56 | H | 0.1 | -- | 4RA0 | G127 | C | -0.01 | -- |
| 1MHP | Y58 | H | -0.5 | -- | 4U6H | N27 | E | -1.204738919 | -- |
| 1MHP | Y59 | H | 0.15 | -- | 4UWQ | R416 | A | -1 | -- |
| 1MHP | L60 | H | -0.12 | -- | 4X4M | K173 | E | -0.75 | -- |
| 1MHP | K64 | H | -0.07 | -- | 4X4M | H174 | E | -0.63 | -- |
| 1MHP | F99 | H | 0 | -- | 4X4M | R175 | E | -1.92 | -- |
| 1MHP | G100 | H | 0.1 | -- | 4Y61 | Y273 | A | -0.044929235 | -- |
| 1MHP | G102 | H | 0.8 | -- | 4Y61 | R114 | B | -0.154916273 | -- |
| 1MHP | A25 | L | 0 | -- | 4YEB | A313 | A | 0.065582469 | -- |
| 1MHP | V29 | L | 0.1 | -- | 4YEB | R117 | B | 0.274118663 | -- |
| 1MHP | H31 | L | 0.3 | -- | 4YEB | F160 | B | -1.687660563 | -- |
| 1MHP | M32 | L | 0.2 | -- | 4YFD | R75 | A | 0.296819816 | -- |
| 1MHP | T50 | L | -0.1 | -- | 4YFD | R196 | A | 0.259762714 | -- |
| 1MHP | L53 | L | -0.4 | -- | 4YFD | D30 | B | -0.024196036 | -- |
| 1MHP | S91 | L | 0.3 | -- | 5C6T | Y280 | A | 3.211234335 | ** |
| 1MHP | G92 | L | 0.2 | -- | 5C6T | N281 | A | 0.284111691 | -- |
| 1MHP | N93 | L | 0.1 | -- | 5C6T | T283 | A | 0.33809636 | -- |
| 1MHP | P94 | L | -0.1 | -- | 5C6T | N284 | A | 0.3614206 | -- |
| 1MLC | N32 | A | 0 | -- | 5C6T | N286 | A | -0.367425539 | -- |
| 1MLC | N92 | A | -1.25 | -- | 5C6T | F297 | A | 2.760639263 | ** |
| 1MLC | T31 | B | 0.13 | -- | 5C6T | R31 | H | 0.311017395 | -- |
| 1MLC | S57 | B | -0.38 | -- | 5C6T | S32 | H | -0.868535725 | -- |
| 1MLC | T58 | B | -0.56 | -- | 5C6T | Y109 | H | 0.3614206 | -- |
| 1MLC | K65 | B | 0.02 | -- | 5C6T | R51 | L | 0.122701557 | -- |
| 1MLC | T28 | B | -0.15 | -- | 5CYK | E481 | A | -0.348404816 | -- |
| 1MTN | T311 | D | 0.2 | -- | 5CYK | T484 | A | -0.646101524 | -- |
| 1MTN | P313 | D | -0.1 | -- | 5CYK | R486 | A | -0.152317007 | -- |
| 1MTN | I319 | D | 0.1 | -- | 5CYK | V488 | A | -0.152317007 | -- |
| 1MTN | R320 | D | 0.3 | -- | 5K39 | L83 | B | -3 | -- |
| 1MTN | V334 | D | 0 | -- | 5E6P | Y1806 | A | 0.364633185 | -- |
| 1MTN | G336 | D | 0.9 | -- | 5E6P | A1832 | A | 0.118505006 | -- |
| 1MTN | R339 | D | 0.2 | -- | 5E6P | A1833 | A | 0.60511707 | -- |
| 1NFI | Y181 | F | S | ** | 5E6P | I66 | B | 0.278580821 | -- |
| 1NFI | C215 | F | N | -- | 5E6P | R88 | B | -0.6 | -- |
| 5K39 | D39 | B | 4 | ** |  |  |  |  |  |

** represents hot spots

-- represents non-hot spots
